# Comprehensive assessment of the genetic characteristics of small for gestational age newborns in NICU: from diagnosis of genetic disorders to prediction of prognosis

**DOI:** 10.1186/s13073-023-01268-2

**Published:** 2023-12-13

**Authors:** Hui Xiao, Huiyao Chen, Xiang Chen, Yulan Lu, Bingbing Wu, Huijun Wang, Yun Cao, Liyuan Hu, Xinran Dong, Wenhao Zhou, Lin Yang

**Affiliations:** 1 Department of Neonatology, Children’s Hospital of Fudan University, National Children’s Medical Center, Shanghai, 201102 China; 2 Center for Molecular Medicine, Children’s Hospital of Fudan University, National Children’s Medical Center, Shanghai, 201102 China; 3 Shanghai Key Laboratory of Birth Defects, Children’s Hospital of Fudan University, National Children’s Medical Center, Shanghai, 201102 China; 4 Guangzhou Women and Children’s Medical Center, Guangzhou Medical University, Guangzhou, 510005 China; 5 Department of Pediatric Endocrinology and Inherited Metabolic Diseases, Children’s Hospital of Fudan University, National Children’s Medical Center, Shanghai, 201102 China

**Keywords:** Small for gestational age (SGA), Newborn, Clinical exome sequencing, Prediction model

## Abstract

**Background:**

In China, ~1,072,100 small for gestational age (SGA) births occur annually. These SGA newborns are a high-risk population of developmental delay. Our study aimed to evaluate the genetic profile of SGA newborns in the newborn intensive care unit (NICU) and establish a prognosis prediction model by combining clinical and genetic factors.

**Methods:**

A cohort of 723 SGA and 1317 appropriate for gestational age (AGA) newborns were recruited between June 2018 and June 2020. Clinical exome sequencing was performed for each newborn. The gene-based rare-variant collapsing analyses and the gene burden test were applied to identify the risk genes for SGA and SGA with poor prognosis. The Gradient Boosting Machine framework was used to generate two models to predict the prognosis of SGA. The performance of two models were validated with an independent cohort of 115 SGA newborns without genetic diagnosis from July 2020 to April 2022. All newborns in this study were recruited through the China Neonatal Genomes Project (CNGP) and were hospitalized in NICU, Children’s Hospital of Fudan University, Shanghai, China.

**Results:**

Among the 723 SGA newborns, 88(12.2%) received genetic diagnosis, including 42(47.7%) with monogenic diseases and 46(52.3%) with chromosomal abnormalities. SGA with genetic diagnosis showed higher rates in severe SGA(54.5% vs. 41.9%, *P*=0.0025) than SGA without genetic diagnosis. SGA with chromosomal abnormalities showed higher incidences of physical and neurodevelopmental delay compared to those with monogenic diseases (45.7% vs. 19.0%, *P*=0.012). We filtered out 3 genes (*ITGB4*, *TXNRD2*, *RRM2B*) as potential causative genes for SGA and 1 gene (*ADIPOQ*) as potential causative gene for SGA with poor prognosis. The model integrating clinical and genetic factors demonstrated a higher area under the receiver operating characteristic curve (AUC) over the model based solely on clinical factors in both the SGA-model generation dataset (AUC=0.9[95% confidence interval 0.84–0.96] vs. AUC=0.74 [0.64–0.84]; *P*=0.00196) and the independent SGA-validation dataset (AUC=0.76 [0.6–0.93] vs. AUC=0.53[0.29–0.76]; *P*=0.0117).

**Conclusion:**

SGA newborns in NICU presented with roughly equal proportions of monogenic and chromosomal abnormalities. Chromosomal disorders were associated with poorer prognosis. The rare-variant collapsing analyses studies have the ability to identify potential causative factors associated with growth and development. The SGA prognosis prediction model integrating genetic and clinical factors outperformed that relying solely on clinical factors. The application of genetic sequencing in hospitalized SGA newborns may improve early genetic diagnosis and prognosis prediction.

**Supplementary Information:**

The online version contains supplementary material available at 10.1186/s13073-023-01268-2.

## Background

Small for gestational age (SGA) is typically defined either as being smaller than the 10th percentile for birth weight at a given gestational age or as having a birth length or weight standard deviation score (SDs) of less than −2.0 [1]. With a prevalence of 6.61% in China, the annual number of SGA births is approximately 1,072,100, making it one of the highest globally [2–4]. Advances in medical technologies and neonatal resuscitation techniques have improved SGA survival rate; however, these survivors continue to be a high-risk population for adverse perinatal outcomes, growth delay, neurocognitive disorders, metabolic disease risk, and adult diseases [5–10]. Consequently, it is crucial to identify disease disorders and predict prognosis for the SGA population.

Genetic factors are believed to account for approximately 46% of the variation in SGA births [11]. Next-generation sequencing (NGS) in neonatal populations serves as an effective tool for characterizing the genetic background of specific patient groups and exploring genetic factors involved in fetal development. Previous genetic studies on SGA populations have mainly concentrated on chromosomal abnormalities, particularly in children born SGA with short stature. Detection rate of copy number variations (CNVs) in these patients varied widely from 9.3 to 58% [12–16]. These variations are mainly due to differing inclusion criteria for cohort subjects. Applications of genetic testing for monogenic and/or chromosomal diseases in the SGA neonatal population exist; however, these studies have been constrained by small sample sizes, with none exceeding one hundred SGA newborns [17, 18].

In addition to pathogenic monogenic diseases and chromosomal abnormalities, the genetic background of the SGA newborns can be further characterized using gene-based rare-variant collapsing analyses and gene burden test to identify potential genetic risk factors. Empirical evidence indicated that rare variants (minor allele frequency < 1%) may represent a novel potential genetic risk factor related to complex diseases [19, 20]. Gene burden analysis posits that all rare variants within a gene or specific region are causal and associated with a trait exerting the same direction and magnitude of effect [21]. By case-control comparison, those genes significantly enriched with rare variants in the case group are likely to be disease risk genes [22–25].

In this study, we retrospectively analyzed the clinical exome sequencing (CES) data of 723 SGA newborns in the newborn intensive care unit (NICU). Our aims were twofold: to investigate the genetic spectrum of SGA newborns with a genetic diagnosis and compare the clinical manifestations associated with different genetic findings, and to identify a set of risk genes for SGA and SGA with poor prognosis by gene-based collapsing analyses and the gene burden test. In addition, a prognosis prediction model was developed. This model incorporated rare variant burden in the identified risk genes and key clinical risk factors and was validated in an independent cohort of 115 SGA newborns. We recognized that our SGA cohort could not be representative of the entire SGA population. To the best of our knowledge, this study represents the first comprehensive assessment on the genetic contributions of monogenic diseases, chromosomal abnormalities, and rare-variant burden in risk genes for SGA on a large scale. The novel prognosis prediction model generated from this study may guide clinical decision-making and improve the management of SGA newborns.

## Methods

### Study participants

**Fig. 1:**
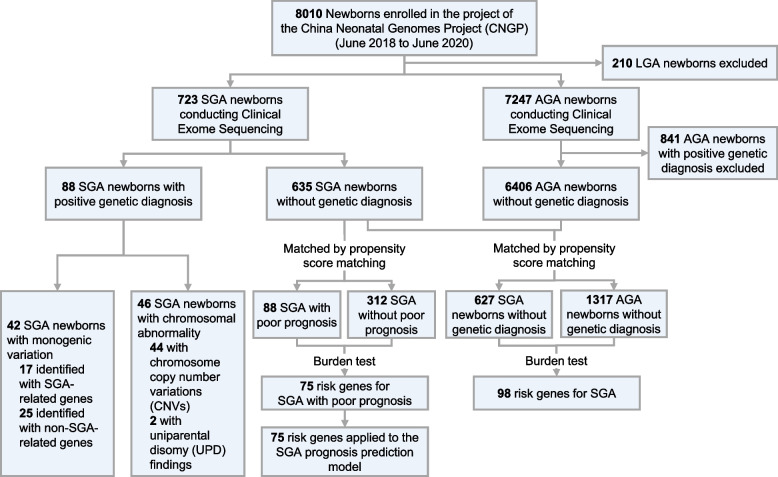
Outline of the study design. SGA indicates small for gestational age. AGA indicates appropriate for gestational age. LGA indicates large for gestational age

Patients participated in this study were recruited through the China Neonatal Genomes Project (CNGP; NCT03931707) [26–28], from NICU of Children’s Hospital of Fudan University, Shanghai, China. The patients included in the CNGP were those who were suspected of having a genetic disorder, and the detailed criteria for inclusion can be found in Additional file 1: Additional method. From June 2018 to June 2020, there were 8010 newborns enrolled in the CNGP. Based on this population, firstly, to describe the genetic spectrum of SGA, we selected 723 SGA newborns from it. Secondly, for exploring the genetic risk factors for SGA, we also selected 7247 AGA newborns from this population, of which 1317 AGA newborns without a genetic diagnosis were selected as controls for subsequent analysis. Moreover, in order to validate the performance of the SGA prognosis prediction model, we enrolled additional 115 SGA newborns without genetic diagnosis from the CNGP between July 2020 and April 2021 (Additional file 2: Figure S1). These SGA newborns were hospitalized in NICU in the same hospital as mentioned above. The study design is illustrated in Fig. 1.

The inclusion criteria for SGA in our study involved newborns with birth weights below the 10th percentile, which was consistent with the recommendations published by the World Health Organization [29], diagnosed by physicians based on the growth standard curves of birth weight for Chinese newborns [30], and patients of the Chinese Han population. AGA inclusion criteria encompassed newborns with a birth weight between the 10th and 90th percentiles, according to the same growth standard curves, and also patients from the Chinese Han population. Patients with trisomy 21 or those unavailable for clinical follow-up information were excluded. This study was approved by the Ethics Committee of the Children’s Hospital of Fudan University (2015-169) and written informed consent from the patients’ parents or legal guardians was obtained for genetic testing and participate in this study.

Clinical information, such as sex, gestational age, birth weight, admission record, discharge diagnosis, and pregnancy information of mothers, was gathered from each newborn’s electronic clinical records. Each newborn’s birth weight was categorized as either less than the third percentile (< P3), or between the third and tenth percentile (P3–P10). Newborns with birth weight < P3 were classified as severe SGA [30, 31]. The clinical information also noted whether the newborn exhibited craniofacial deformities, central nervous system anomalies, cardiovascular abnormalities, evidence of metabolic disease, digestive system anomalies, respiratory system anomalies, skeletal abnormality, urinary or reproductive system, infection and immune involvement, hematologic abnormalities, and congenital malformation during the neonatal period. All clinical information is recorded by experienced neonatologist with a strong clinical genetics background. Detailed phenotypes for each organ system abnormality are shown in Additional file 1: Additional method.

Mother’s pregnancy information comprised details like abnormalities of the placenta, amniotic fluid, umbilical cord at birth, complications during pregnancy, maternal age, and whether in vitro fertilization and embryo transfer (IVF-ET) technology was utilized for this pregnancy. We also recorded results of several maternal obstetrical examinations. These included detection of intrauterine growth retardation (IUGR) or fetal growth restriction (FGR) during pregnancy, observation of fetal distress, high-risk suggestions by noninvasive DNA or amniocentesis, and detection of structural malformations (brain, heart, bone, digestive system) during pregnancy. However, due to the retrospective nature of this study, the information regarding maternal obstetric examinations relying on neonatal clinical records in the NICU may be incomplete.

Each SGA newborn in this study was followed up for over 2 years, and any physical growth delay, neurodevelopmental delay, or death was documented, with any of these outcomes classified as a poor prognosis. Evaluation and diagnosis of developmental delay were determined by physicians from the Division of Child Health Care. Physical growth delay indicated the length falling below the 3rd percentile of expected growth [32], and neurodevelopmental delay indicated by an F quotient < 75, detected by Gesell Developmental Schedules.

### Clinical exome sequencing and variant annotation

Clinical exome sequencing (CES) was performed on each enrolled newborn. Genomic DNA samples were extracted from whole blood using a QIAamp DNA Blood Mini Kit (Qiagen, Hilden, Germany). DNA fragments were enriched for CES using the Agilent ClearSeq Inherited Disease Kit (Agilent Technologies, Santa Clara, CA) covering 3203 genes, which included 2742 confirmed disease-causing genes [33, 34]. Sequencing was conducted on an Illumina HiSeq 2000/2500 platform (Illumina, San Diego, CA, USA). Low-quality reads (reads containing more than 10% unknown bases or more than 50% bases with a sequencing quality of < 5) were removed from the raw fastq data to generate clean reads. Clean reads were aligned to the reference human genome (University of California, Santa Cruz [UCSC] hg19) using the Burrows-Wheeler Aligner (BWA; v.0.5.9-r16), sorted by SAMtools (v.1.8), and deduplicated using Picard (v.2.20.1). The average on-target sequencing depth was at least 100×. For variant calling, Genome-Analysis-Toolkit best practice (V.3.2) was employed for single-nucleotide variations (SNVs)/small indels, and CANOES and HMZDelFinder were separately applied to detect CNVs, and the results were then merged. Details of the CNGP clinical sequencing pipeline have been described in our published article [35].

We conducted a genetic analysis of the candidate variants following the criteria set by the American College of Medical Genetics and Genomics (ACMG) guidelines and ClinGen Sequence Variant Interpretation guidelines [36–39]. All diagnostic SNVs/small indels were confirmed in the proband and parents, if available, using Sanger sequencing. Primers were designed for polymerase chain reaction (PCR) amplification using Primer Premier 5.0 software. Sequence analysis was performed using MutationSurveyor software (SoftGenetics, State College, PA, USA). Only pathogenic or likely pathogenic variants were reported. We annotated and filtered detected CNVs, considering known pathogenicity, variant size, and the genes affected by the CNV [40, 41]. The Bcftools/RoH method was used to determine loss of heterozygosity (LOH), which is suggestive of potential uniparental disomy (UPD) [42]. The detected LOH was filtered based on its location in a region associated with growth failure (Additional file 1: Table S1). Methylation-specific multiplex ligation-dependent probe amplification (MS-MLPA) verification was performed for the detected LOH or deletions encompassing the 15q11-q13 Prader-Willi/ Angelman critical regions.

### Gene-based collapsing analyses and gene burden test

We test if having gene variant information would provide additional prediction power to SGA. We selected SGA and AGA samples without a genetic diagnosis for analyses. First, we selected 627 SGA newborns as the case group, and 1317 AGA newborns as the control group for the gene burden test; these two groups were matched for gestational age, sex, and whether the mother had gestational hypertension through propensity score matching (PSM). Secondly, we chose 88 SGA newborns with poor prognosis as the case group, and 312 SGA newborns without poor prognosis as the control group for gene burden test; these two groups were also matched for gestational age, sex, and gestational hypertension of mothers by PSM. The workflow of gene burden test is displayed in Fig. 1. The cases-control pairing by PSM was provided in the Additional file 1: Table S2.

To prepare for the rare-variant collapsing analyses, we took the following steps. First, each variant in the selected newborns was classified into four variant types as protein-truncating variants (PTVs), missense or non-synonymous variants (MISs), synonymous variants (SYNs), and non-coding variants (NONs) [43]. Next, for each gene in each sample, the number of variants for each type was calculated and summarized. Then, we applied Fisher’s exact test to examine whether the number of each variant type was significantly higher in the case group than in the control group. Synonymous variants and non-coding variants were treated as a near-neutral background, and genes with significant differences found at the synonymous or non-coding level were filtered out. The details of the gene-based collapsing analyses pipeline have been described in our published work [34]. Risk genes with *P*_*PTV*_ <0.05 or *P*_*MIS*_ < 0.05 are retained for subsequent analysis.

By combining the significance of PTV and MIS variants from the gene burden test, we developed a gene scoring system to select risk genes. The risk score for each gene was defined as follows:

$$Risk\ Score=2\ast \left(-{\mathit{\log}}_{10}\left({P}_{PTV}\right)\right)+\left(-{\mathit{\log}}_{10}\left({P}_{MIS}\right)\right)$$

We built the null distribution of the expected risk score of each gene by performing 10,000 permutation tests. Each time we randomly shuffled the original case-control labels of the sample, and each time used the shuffled case-control labels to recalculate the risk score. The permutation *P* value for each gene was computed by testing whether the observed combined risk score was significantly higher than the null distribution. Here, correction for *P* values is indeed essential in multiple testing of hypotheses but Bonferroni method is too conservative. We applied false discovery rate (FDR) method to correct the permutation *P* value. The genes with *P*_*PTV*_ <0.01 or *P*_*MIS*_ < 0.01, combining with FDR of permutation *P* value <0.01 were defined as potential causative genes [44].

### SGA prediction model generation and validation

Building on the risk genes for SGA with poor prognosis, each newborn was assigned a rare-variant burden score as the genetic predictors, which was noted as 2 for carrying PTVs of risk genes, 1 for carrying the MISs of risk genes, or 0 for carrying other variant types of risk genes. We selected 627 SGA newborns as the SGA-model generation dataset, samples from this dataset were subsequently divided into training and testing datasets at a 7:3 ratio through random sampling. In the training dataset, we selected the significant clinical phenotypes observed during neonatal hospitalization using univariable logistic regression as clinical factors. Following this, we utilized the Gradient Boosting Machine (GBM) framework to generate two prediction models to anticipate the prognosis of SGA, one incorporating only the selected clinical factors, and the other encompassing both the selected clinical factors and genetic predictors. A 10-fold cross-validation strategy was used for optimal parameter selection, with three complete sets of folds computed. The performance in the testing dataset was used to evaluate the performance of the above two models using the area under the receiver operating characteristic curve (AUC).

Furthermore, we enrolled 115 SGA newborns without genetic diagnosis by CES as an independent SGA-validation dataset for model validation. The two prediction models were applied to predict the prognosis of each SGA newborn, with the AUC also applied to evaluate the performance of the two prediction models in the SGA-validation dataset. The workflow of generating and validating the SGA prediction model is presented in the Additional file 2: Figure S1.

### Statistical analysis

Continuous data were described using means and standard deviations. Differences in initial clinical characteristics, follow-up characteristics, and maternal information between the two groups were analyzed using the *t*-test or Wilcoxon rank sum test for continuous variables, and the chi-square test or Fisher’s exact test for categorical variables. The multivariate logistic regression analysis was performed to estimate the association between genetic diagnosis and a set of predictor variables, including gestational age, sex, and maternal gestational hypertension. PSM was applied to the SGA and AGA samples using the R package MatchIt. The Pearson correlation coefficient was used for gene expression correlation analysis. The *P* values of multiple comparisons were adjusted by the FDR. All statistical analyses were conducted using the R package (V.4.2.1).

## Results

### Clinical characteristics of SGA patients

**Fig. 2:**
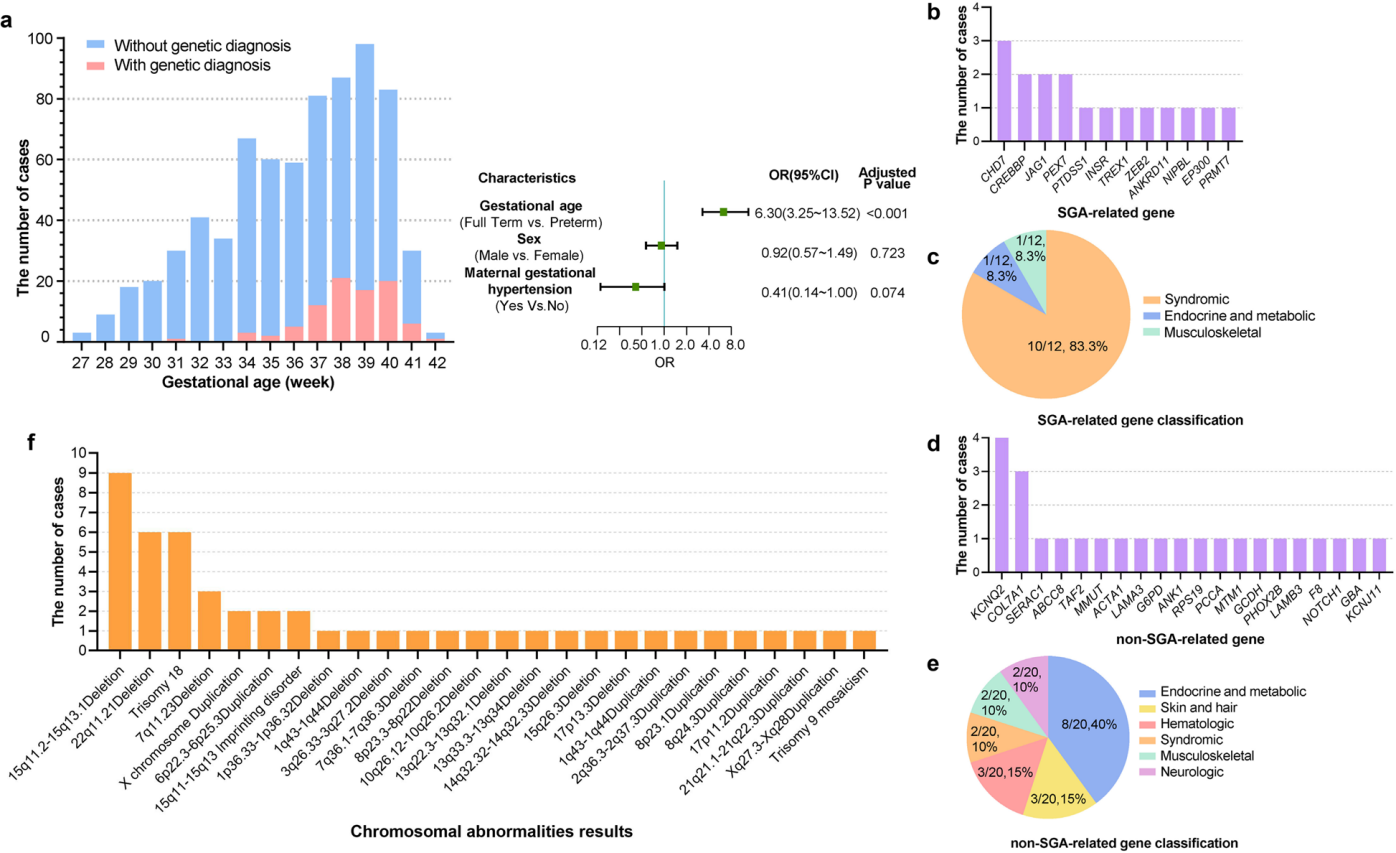
The distribution of gestational age and genetic findings in our 723 SGA newborns. **a** The distribution of gestational age. **b** The number of newborns with SGA-related genes detected. **c** The classification of SGA-related genes according to the main phenotype of related diseases. **d** The number of newborns with non-SGA-related genes detected. **e** The classification of non-SGA-related genes according to the main phenotype of related diseases. **f** The number of newborns with chromosomal abnormality detected

A total of 723 (439 males [60.7%] and 284 females [39.3%]) SGA newborns were included in this study. The mean gestational age and birth weight were 36.5 weeks and 2002.1 g, respectively (Table 1). The SGA population spanned gestational ages from 27 to 42 weeks, with the majority being term births (37–42 weeks, 52.42%). Extremely preterm births (< 28 weeks) and post-term births (> 42 weeks) represented the smallest proportions (0.41% each). The logistic regression analysis revealed a significantly higher likelihood of a genetic diagnosis for full-term births (gestational age≥37 weeks) compared to preterm births (gestational age <37 weeks) (OR=6.30, 95%CI 3.25–13.5; *P*<0.001) (Fig. 2a). The rate of severe SGA was greater in patients with a genetic diagnosis (54.5% vs. 41.9%, *P* = 0.0025) than in those without. Follow-up results showed that SGA newborns with genetic diagnosis exhibited worse prognoses, demonstrating higher rates of death (19.3% vs 5.4%, *P* < 0.0001), growth delay (31.9% vs 4.1%, *P* < 0.0001), and neurodevelopmental delay (31.9% vs 5.9%, *P* < 0.0001).

**Table 1:** Characteristics of the SGA newborns in this study

	All SGA newborns (*n* = 723)	SGA newborns with genetic diagnosis (*n* = 88)	SGA newborns without genetic diagnosis (*n* = 635)	*P* value
**Birth information**				
Male (%)	439 (60.7)	52 (59.1)	387 (60.9)	0.74
Gestational age, mean (SD), week	36.5(3.3)	38.7(1.8)	36.2(3.3)	< 0.00001
Birth weight, mean (SD), g	2002.1(626.8)	2362.5(424.8)	1952.2(634.1)	< 0.00001
Severe SGA (%)	314 (43.4)	48 (54.5)	266 (41.9)	0.025
**Organ system abnormalities during hospitalization**				
Cardiovascular (%)	515 (71.2)	59 (67.0)	456 (71.8)	0.36
Allergy/immunologic/infectious (%)	346 (47.9)	39 (44.3)	307 (48.3)	0.48
Hematologic (%)	271 (37.5)	25 (28.4)	246 (38.7)	0.061
Metabolic/biochemical (%)	264 (36.5)	27 (30.7)	237 (37.3)	0.23
Neurologic (%)	228 (31.5)	41 (46.6)	187 (29.4)	0.0012
Gastrointestinal (%)	216 (29.9)	19 (21.6)	197 (31.0)	0.070
Respiratory (%)	212 (29.3)	22 (25.0)	190 (29.9)	0.34
Renal/genital (%)	91 (12.6)	16 (18.2)	75 (11.8)	0.091
Skeletal (%)	50 (6.9)	15 (17.0)	35 (5.5)	0.000064
Craniofacial (%)	39 (5.4)	8 (9.1)	31 (4.9)	0.10
Dermatologic (%)	29 (4.0)	6 (6.8)	23 (3.6)	0.15
Congenital structural malformation (%)	319 (44.1)	51 (58.0)	268 (42.2)	0.0053
**Follow-up characteristics**				
Death (%)	51 (7.1)	17 (19.3)	34 (5.4)	< 0.00001
Developmental delay (%)	99 (13.7)	39 (44.3)	60 (9.4)	< 0.00001
Growth delay (%)	55 (7.6)	29 (31.9)	26 (4.1)	< 0.00001
Neurodevelopmental delay (%)	66 (9.1)	29 (31.9)	37 (5.9)	< 0.00001

Maternal and pregnancy information were also considered since maternal factors could impact SGA births (Table 2). Compared with mothers of SGA newborns with genetic findings, mothers of SGA newborns without genetic findings had higher rates of gestational hypertension (6.0% vs 25.9%, *P* = <0.001) and of gestational diabetes mellitus (8.4% vs 18.4%, *P* = 0.024). As this retrospective study focused on hospitalized neonatal cases, information on maternal prenatal examinations was limited. Among documented prenatal information, 78.1% (157/201) of the mothers had recorded fetal distress; IUGR/FGR and fetal structural malformations of the brain, heart, bones, or digestive system were reported for 97.3% (146/150) and 42.6% of pregnancies, respectively. Of the 46 mothers who underwent noninvasive DNA testing, 4 (8.7%) received high-risk results.

**Table 2:** Maternal characteristics of the SGA newborns in this study

	All SGA newborns (*n* = 723)	SGA newborns with genetic diagnosis (*n* = 88)	SGA newborns without genetic diagnosis (*n* = 635)	*P* value
**Fetal appurtenances information**				
Fetal appurtenances abnormality (%) *				0.42
Yes	267 (38.1)	35 (42.2)	232 (37.6)	
No	433 (61.9)	48 (57.8)	385 (62.4)	
NA	23	5	18	
Placental abnormalities (%)				0.50
Yes	47 (6.7)	7 (8.4)	40 (6.5)	
No	653 (93.3)	76 (91.6)	577 (93.5)	
NA	23	5	18	
Amniotic fluid abnormalities (%)				0.87
Yes	197 (28.1)	24 (28.9)	173 (28.0)	
No	503 (71.9)	59 (71.1)	444 (72.0)	
NA	23	5	18	
Umbilical cord abnormalities (%)				0.32
Yes	71 (10.1)	11 (13.3)	60 (9.7)	
No	629 (89.9)	72 (86.7)	557 (90.3)	
NA	23	5	18	
**Complication of pregnancy**				
Diabetes mellitus (%)				0.024
Yes	123 (17.2)	7 (8.4)	116 (18.4)	
No	592 (82.8)	76 (91.6)	516 (81.6)	
NA	8	5	3	
Hypertension (%)				<0.001
Yes	169 (23.6)	5 (6.0)	164 (25.9)	
No	548 (76.4)	78 (94.0)	470 (74.1)	
NA	6	5	1	
Thyroid disease (%)				0.72
Yes	59 (8.2)	6 (7.2)	53 (8.4)	
No	657 (91.8)	77 (92.8)	580 (91.6)	
NA	7	5	2	
Intrahepatic cholestasis (%)				0.10
Yes	22 (3.1)	0 (0)	22 (3.5)	
No	694 (96.9)	83 (100.0)	611 (96.5)	
NA	7	5	2	
Infection (%)				0.60
Yes	72 (10.1)	7 (8.4)	65 (10.3)	
No	644 (89.9)	76 (91.6)	568 (89.7)	
NA	7	5	2	
**Maternal characteristics**				
Maternal age, mean (SD), years old	30.22 (5.05)	29.72 (5.69)	30.28 (4.96)	0.26
Maternal age >35 years old (%)				0.47
Yes	143 (20.1)	19 (23.2)	124 (19.7)	
No	567 (79.9)	63 (76.8)	504 (80.3)	
NA	13	6	7	
In vitro fertilization and embryo transfer (IVF-ET) (%)				0.12
Yes	85 (11.8)	79 (6.8)	6 (12.4)	
No	638 (88.2)	556 (93.2)	82 (87.6)	

* Fetal appurtenances abnormality refers to abnormalities of the placenta or amniotic fluid or umbilical cord observed at birth. The placental abnormalities include morphological abnormalities (small placenta, large placenta, battledore placenta, velamentous placenta, etc.) and functional abnormalities (placental degeneration, necrosis, infarction, aging). Amniotic fluid abnormalities include abnormal amniotic fluid volume (polyhydramnios or oligohydramnios), hemorrhagic amniotic fluid, and amniotic fluid contamination of II° and above. Umbilical cord abnormalities include morphological abnormalities (thin, spiral, etc.) umbilical cord around the neck more than one circle, single umbilical artery

NA: not available

Regarding the clinical phenotypes during hospitalization, the three most affected systems were cardiovascular (515/723, 71.2%), immune (346/723, 47.9%), and blood systems (271/723, 37.5%). SGA newborns with genetic diagnosis were more likely to display abnormalities in the neurologic (46.6% vs. 29.4%, *P*=0.0012), skeletal abnormalities (17.0% vs. 5.5%, *P*=0.000064), and congenital structural malformation (58.0% vs. 42.2%, *P*=0.0053) during the neonatal period than those without a genetic diagnosis. Aside from these specific organ systems, there were no significant differences in abnormalities in other systems between patients with and without a genetic diagnosis.

### SGA with genetic diagnosis

In total, 88 SGA newborns received a genetic diagnosis, including 42 patients (47.7%) with monogenic diseases and 46 patients (52.3%) with chromosomal abnormalities, leading to an overall genetic diagnosis rate of 12.2% (88/723) [45]. When comparing these two groups with different genetic diagnosis, no significant differences were observed in severe SGA. However, growth delay (45.7% vs. 19.0%, *P* = 0.012) and neurodevelopmental delay (45.7% vs. 19.0%, *P* = 0.012) are more prevalent in the chromosomal abnormality than in the monogenic disease groups. The combined occurrence of growth and neurodevelopmental delay is further pronounced in the chromosomal abnormality group (37.0% vs. 4.8%, *P* = 0.00021) (Additional file 1: Table S3).

Accounting for follow-up information, SGA patients with physical growth delay were classified as SGA-short patients, and the genetic diagnosis rate for SGA-short was 52.7% (29/55). During the neonatal period, SGA-short with a genetic diagnosis presented a higher prevalence of severe SGA than SGA-short without genetic findings (72.4% vs. 34.6%, *P *= 0.0049). Among the 29 SGA-shorts with a genetic diagnosis, 8 (27.6%) had monogenic disease and 21 (72.4%) had chromosomal abnormalities, indicating a threefold higher detection rate for chromosomal abnormalities compared to monogenic disease.

### Monogenic disease results

Regarding the monogenic disease findings, 32 genes were identified across 42 patients. Genes causing diseases that affect intrauterine and bone development, or those previously reported to be associated with SGA were classified as SGA-related genes (Additional file 1: Table S4), whereas all others identified genes were classified as non-SGA-related genes (Additional file 1: Table S5). Based on the primary phenotypes of the associated diseases, these identified genes were further divided into six categories: Syndromic (gene resulting in a syndrome that affects multiple organs or systems; Additional file 1: Table S6); Endocrine and metabolism-related; Musculoskeletal; Skin and hair-related; Hematologic; and Neurologic (Fig. 2c, e and Additional file 1: Table S7).

Among the identified SGA-related genes, the majority (10/12, 83.3%) were syndromic genes, with *CHD7* being the most recurrent gene (identified in three cases; Fig. 2b, c). On the other hand, the most commonly identified non-SGA-related gene was *KCNQ2* (occurring in four cases; Fig. 2d). Disorders caused by non-SGA-related genes were distributed across different organ systems (Fig. 2e). Newborns with SGA-related genes had a higher proportion of severe SGA (70.6% vs. 32.0%, *P* =0.027) and physical growth delay (35.3% vs. 8.0%, *P* =0.045) than those with non-SGA-related genes, while no significant differences in terms of death and neurodevelopmental delay between the two groups (Additional file 1: Table S8).

### Chromosomal abnormalities

Chromosomal abnormalities observed in the study population included 25 CNV and 1 UPD findings detected across 46 patients (Additional file 1: Table S9). Regarding CNV findings, 25 CNVs, comprising 14 deletions, 8 duplications, and 3 karyotype abnormalities, were detected in 44 patients. High-frequency CNV findings included 15q11-q13 deletion in 9 cases, 22q11.21 deletion in 6 cases, and 7q11.23 deletion in 3 cases (Fig. 2f), which correspond to Prader–Willy syndrome (PWS, confirmed by MS-MLPA), DiGeorge syndrome, and Williams syndrome, respectively. Nine patients were detected with karyotype abnormalities, with trisomy 18 being the most common, detected in six patients.

Two males with UPD findings presented increased methylation in 15q11-q13. Both of them presented with muscular hypotonia, poor feeding, and cryptorchidism during the neonatal period. Combining their clinical information with genetic findings, both were diagnosed with PWS caused by maternal UPD 15. Therefore, PWS was the most frequent (11/46, 23.9%) chromosomal disorder in this study.

### Risk genes for SGA and SGA with poor prognosis

In our gene burden test comparing SGA vs. AGA samples, a total of 98 genes were identified as the risk genes for SGA. In another gene burden test comparing SGA with poor prognosis vs. SGA without poor prognosis, 75 genes were identified as the risk genes for SGA with poor prognosis (Additional file 1: Table S10).

At significance of *P*_*PTV*_ <0.01 or *P*_*MIS*_ < 0.01, combined with FDR of permutation process <0.01, there were 3 potential causative genes for SGA, including *ITGB4*, *TXNRD2*, and *RRM2B*. Only one gene *ADIPOQ* was filtered out as a potential causative gene for SGA with poor prognosis (Additional file 1: Table S10).

### SGA prognosis prediction model

Among SGA patients without a genetic diagnosis, 14.3% (91/635) had a poor prognosis. To predict the risk of SGA with poor prognosis, we employed a machine learning model incorporating clinical and genetic risk factors.

**Fig. 3:**
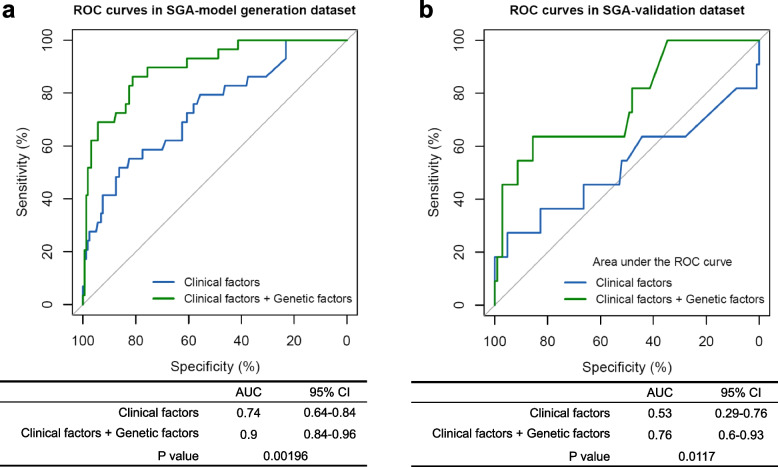
Receiver operating characteristic (ROC) analyses of predictive models for SGA prognosis. Clinical factors were selected by the univariable logistic regression, including the significant different clinical phenotypes between SGA with poor prognosis and SGA without poor prognosis during neonatal hospitalization. Genetic factors included the rare-variant burden score for the 75 risk genes of SGA newborns with poor prognosis. **a** ROC curves in SGA-model generation dataset. **b** ROC curves in SGA-validation dataset

Six clinical factors, including neurologic abnormalities (OR=3.1, 95% confidence interval [CI] 1.96–4.92; *P* < 0.0001), metabolic/biochemical abnormalities (OR=2.22, 95%CI 1.41–3.5; *P*= 0.00058), skeletal abnormalities (OR=3.25, 95%CI 1.35–7.32; *P* = 0.0056), respiratory abnormalities (OR=2.6, 95%CI 1.65–4.11; *P*< 0.0001), allergy/immunologic/infectious (OR=2.01, 95%CI 1.28–3.23; *P*= 0.0030), and craniofacial abnormalities (OR=3.31, 95%CI, 1.49–6.96; *P*= 0.0021), were significantly different between the SGA with and without poor prognosis in SGA-model generation dataset (SGA without genetic diagnosis included in gene burden test, *n*=627). The prediction model using only these six clinical factors yielded an AUC of 0.74 (95%CI 0.64–0.84). In contrast, the model that combined six clinical factors with the genetic predictors improved the AUC to 0.9 (95%CI 0.84–0.96), demonstrating significantly better performance (*P*=0.00196, Fig. 3a).

The efficacy of the prediction models was tested using an SGA-validation dataset of 115 SGA newborns without a genetic diagnosis. The baseline information for the SGA-model generation dataset (*n*=627) and the SGA-validation dataset (*n*=115) are available in the Additional file 1: Table S11. No significant difference (*P* = 0.18) in the proportion of poor outcome was observed between the SGA-model generation dataset (14.2%, 89/627) and in the SGA-validation dataset (9.6%, 11/115). In SGA-validation dataset, the prediction model utilizing only six clinical factors presented an AUC of 0.53 (95%CI 0.29–0.76), while the other model that combined six clinical factors with the genetic predictors achieved an AUC of 0.76 (95%CI 0.6–0.93), demonstrated improved accuracy and superior performance (*P* = 0.0117, Fig. 3b).

## Discussion

The etiology of SGA is heterogenous, encompassing environmental, parental, and placental factors, and importantly, genetic factors [11, 46–48]. Many monogenic diseases and genetic syndromes leading to low birth weight, short stature, and growth retardation have been reported. NGS can serve as an effective tool to characterize the genetic landscape with the potential to optimize interventions for the SGA population.

There have been several reports combining pathogenic gene variants and chromosomal abnormalities in SGA populations: Stalman et al. examined 21 SGA and 24 AGA newborns and identified three CNVs, one systematically disturbed methylation pattern, and one sequence variant explaining SGA [17]. Hara-Isono et al. scrutinized 86 SGA children with short stature but without imprinting disorders, and identified 8 (9.3%) and 11 (12.8%) patients with pathogenic CNVs and candidate pathogenic variants, respectively [14]. Peeters et al. evaluated 20 SGA children with short stature treated with growth hormone and identified likely pathogenic variants in 4 children, pathogenic CNVs in 2 probands, and one DNA methylation signature in a child harboring an NSD1-containing microduplication [16]. It is important to note, however, that these published studies primarily focused on Caucasian patients from developed countries and with limited in sample sizes. Therefore, these results may not be generalizable to the SGA population in developing countries with high SGA births such as China.

Our study leveraged a larger cohort of 723 hospitalized SGA newborns of Chinese Han population with an overall genetic diagnosis rate of 12.2%, and genetic findings comprised 47.7% monogenic diseases and 52.3% chromosomal abnormalities. Among the 55 SGA-shorts patients, 52.7% received a genetic diagnosis, 27.6% of which were diagnosed with monogenic diseases and 72.4% for chromosomal abnormalities. To our knowledge, this is the first large-scale study to comprehensively assess the genetic background of hospitalized SGA newborns. Our results demonstrated that monogenic diseases and chromosomal abnormalities each accounted for approximately one-half of the genetic diagnoses, which may provide a more complete distribution description of hospitalized SGA newborns’ genetic backgrounds.

The gestational age distribution in the SGA newborns in this study was similar to that of a prior study on Chinese hospitalized SGA newborns [2], covering all birth types from extremely preterm to post-term. Previous research on the genetic diagnosis rate of rare pediatric disease [49] also showed that probands born prematurely (OR 0.73, 95% CI 0.64–0.82) had a lower likelihood of receiving a genetic diagnosis. For SGA newborns, those born preterm had a lower incidence of genetic diagnosis compared to full-term SGA. This disparity may be due to premature neonates more often requiring NICU admission for complications stemming from immature organ development. In addition, phenotypes of genetic disorders may be concealed in preterm infants due to immature organ development, whereas in full-term infants, abnormal phenotypes may be more noticeable, prompting genetic testing to clarify the cause.

The proportion of severe SGA was elevated in SGA newborns with genetic diagnosis, indicating a greater impact of genetic factors on birth weight. Clinical manifestations in this cohort suggested that when SGA newborns with neurological and skeletal malformations, clinicians should consider potential genetic contributors for these abnormalities and may benefit from performing NGS. Follow-up data showed that higher rates of developmental delay in SGA newborns with genetic diagnosis, especially in SGA newborns with chromosomal abnormalities. A combination of the clinical data from the neonatal period and prognosis information revealed that genetic factors significantly impact the severity of intrauterine and extrauterine growth failure in SGA newborns. Chromosomal abnormalities were observed to exert a more pronounced effect on postnatal development, highlighting the need for early, comprehensive intervention for affected individuals.

Regarding the results for monogenic diseases, SGA-related genes had a greater impact on SGA severity and physical growth. It primarily encompassed syndromic genes, potentially affecting the fundamental regulatory pathways of early organismic development, resulting in multifaceted dysfunctions across multiple systems. In this study population, three patients carrying *CHD7* variants had severe nervous and circulatory systems phenotypes after birth, accompanied by congenital deformities and ultimately leading to death. Non-SGA-related genes, although not currently associated with abnormal intrauterine development, may still affect postnatal development albeit not as typically as syndrome-related genes. Their implications for development may be reflected in metabolic disorders that occur with age and gradually lead to physical developmental delay in childhood, whereas their implications for fetal development remain uncertain. While our classification of genes relies on current literature, the variants in non-SGA-related genes could feasibly serve as plausible candidates for causality, and further investigations may clarify their potential impact on intrauterine or postnatal development.

The most frequent genetic etiology identified was PWS, occurring in ~1/66 (11/723) SGA newborns in our cohort and showing a 150X enrichment compared to the general population (~1/10,000). Identified PWS genetic lesions included nine cases of 15q11-q13 deletions and two cases of maternal UPD 15. In the etiology of PWS patients, paternal deletion accounts for 65–75% and maternal UPD accounts for 20–30% [50]; the etiological distribution of PWS patients in our study was consistent with this proportion. The typical PWS phenotype in the neonatal period is severe hypotonia [50], whereas no causal relationship between PWS and SGA has been clearly proven for birth weight. Published studies have indicated that approximately 50% of PWS patients were SGA [51], possibly because of the central role of epigenetics and imprinted genes in placental development and function [52]. Previous literature has shown that paternally expressed genes in the human placenta promote the extraction of resources from the mother to boost fetal and postnatal growth, while maternally expressed genes inhibit fetal and postnatal growth to conserve maternal resources. PWS, marked by a lack of paternally expressed genes, may favor limiting fetal growth to protect maternal resources [52]. Our results complement studies on the relationship between PWS and SGA, supporting that PWS may be one of the most prevalent chromosomal abnormalities in the SGA population.

Most SGA newborns are expected to experience a period of accelerating growth during the first 2 years of life [53]. However, not all SGA newborns can manage to catch up to normal growth, especially those born very prematurely and with more severe degrees of growth retardation. In addition, catch-up growth may be incomplete in SGA newborns with genetic disorders [1]. Given the association of cognitive impairment with low birth weight [54], published management strategies for SGA [1] recommended early and continuous growth surveillance, and early neurodevelopment evaluation and interventions in at-risk children. Our SGA cohort, derived from NICU newborns, contains preterm infants and patients with multisystem involvement more prone to adverse developmental outcomes. Genetic screening has been previously recommended for SGA children with short statue, and genetic screening strategies could potentially increase the safety of recombinant human growth hormone (rhGH) therapy [55]. Our results demonstrated the value of early-stage genetic testing for NICU SGA infants in detecting the genetic background, with NGS data facilitating timely and precise treatment interventions to improve patient outcomes.

There was also another definition of SGA, which refers to the newborns having a birth weight standard deviation score (SDs) of less than −2.0. When adopting this 2SD definition as the inclusion criteria, the number of SGA newborns with a positive genetic diagnosis would reduce from 88 to 43. Among the 45 excluded patients, there were 5 cases of DiGeorge syndrome, 5 cases of PWS, and 2 cases of CHARGE syndrome due to the *CHD7* gene. These excluded newborns with genetic diagnoses continue to exert potential impact on intrauterine development. Moreover, the overall enrollment of SGA newborns in this study would decrease from 723 to 262, a total of 461 SGA newborns would be excluded from the overall study population, 82 of whom had a poor prognosis, 56 of whom had physical growth delay or (and) neurodevelopmental delay. There were potential genetic factors that could have affected their intrauterine development, and potentially resulted in postnatal developmental abnormalities, which might have been detectable as early as the neonatal period. If we adopt the stricter 2SD definition as SGA criteria may result in the absence of this type of developmental information, potentially leading to missed opportunities for early intervention among several patients who require specific attention for growth catch-up. Therefore, when considering the two distinct definitions of SGA, the 10th centile definition appears to be more effective to identifying a genetic cause compared to the strict -2SD definition. And we have maintained the definition of SGA as less than 10th percentile, which recommended by the WHO and commonly used in clinical practice in China.

In addition to describing the genetic spectrum of SGA newborns, we also systematically investigated the potential genetic etiologies in SGA and in SGA with poor prognosis through rare-variant collapsing analysis and in silico functional interaction analysis. The gene burden test is a popular strategy used to detect genetic risk for disease. Unlike genetic diagnosis, the relationship between genes detected by burden test and phenotypes were more about association rather than determinism [22–25]. This method allows for preliminary analysis based on sequencing dataset to explore genetic risk and disease mechanisms. For example, Lange et al. utilized the gene burden test to investigate the relationship between rare variants and low-density lipoprotein cholesterol (LDL-C) levels, revealing the novel association of the *PNPLA5* gene with an increase in LDL-C [23]. In our study, we made an initial attempt to apply the gene burden test in SGA population to find genetic risk related to SGA and genetic risk related to SGA with poor prognosis.

We filtered out 3 genes (*ITGB4*, *TXNRD2*, *RRM2B*) as potential causative genes for SGA and 1 gene (*ADIPOQ*) as potential causative gene for SGA with poor prognosis. The *ADIPOQ* gene encodes adiponectin that circulates in the plasma and is involved with metabolic and hormonal processes, adiponectin gene knockout mice had known to suffer developmental failure phenotypes such as abnormal growth, increased energy expenditure, decreased fat content, and lower body weight [56, 57]. Thus abnormalities in this gene lead to postnatal developmental delay, which is consistent with our finding that it is a potential causative gene for SGA with poor prognosis. The *ITGB4* gene encodes the integrin beta 4 subunit, integrin beta-4 signaling has been reported to play a pivotal role in embryogenesis, knock-in mice with targeted deletion of beta 4-integrin showed had smaller litter sizes and lower fecundity rate, and the embryos demonstrated a high degree of fragmentation and asymmetry, with fewer surviving to either a morula or blastocyst stage [58]. The protein encoded by the *TXNRD2* gene is a member of the thioredoxin system and plays a crucial role in redox homeostasis. Mice homozygous for a knockout allele die at embryonic day 13 due to severe anemia and growth retardation [59]. And the protein encoded by the *RRM2B* gene is of key importance in cell survival by repairing damaged DNA. Loss of both functional copies of this gene results in growth retardation, multiple organ failure, and ultimately premature death [60, 61]. Abnormalities in these three genes, which all have been published as involving embryonic developmental restriction, support the result that we found them to be the potential causative genes for SGA. Though we cannot directly treat them as the definite causative factors for SGA, their contribution to SGA worth further exploration by including more SGA samples and functional studies. Our findings indicating that the rare-variant collapsing analyses studies have the ability to identify potential causative factors associated with growth and development, and further functional or cohort research on these findings can be warranted.

Given the ongoing clinical concern about the futural development of SGA newborns, we developed a predictive model for SGA prognosis using genetic risk factors detected from the from burden test combined with clinical factors in the neonatal period. The use of genetic risk factors significantly increases the AUC in two independent datasets, especially in the independent validation dataset, proving that our prediction model has an effective prediction effect, and suggesting that a few categories of clinical information and patients’ sequencing data could classify the probable prognosis of SGA. This approach has clinical significance in facilitating early diagnosis of SGA neonates with poor prognoses and optimizing clinical management. Furthermore, it provides a valuable reference for predicting the future development trajectory of SGA as early as the neonatal period. Such information aids in the timely growth hormone therapy and rehabilitation treatment and may positively impacts on treatment compliance for patients and their families.

Our study had several limitations in providing an accurate understanding of the genetic landscape of SGA. Firstly, our study cohort consisted of newborns hospitalized in the NICU, lacked complete maternal obstetric examination information, and genetic findings, based on hospitalized SGA newborns, may not extend to the general SGA population without a larger sample size and more systematic design. Secondly, we used CES rather than WES or WGS for genetic testing, potentially introducing analytical bias due to potential missed genetic diagnoses. Additionally, we did not perform a comprehensive methylation analysis or imprinting disorder screening, indicating a need for further research for imprinting disorders in our SGA patients. Lastly, we do not present secondary findings in this article. Our research group previously published an article demonstrating the detection of secondary findings in neonates from the CNGP [62]. In the future, we plan to continue exploring secondary findings in all neonates enrolled in the CNGP cohort.

## Conclusions

In conclusion, our study provides a comprehensive overview of the genetic findings from a substantial SGA newborn cohort in NICU. In those SGA newborns with a genetic diagnosis, monogenic diseases and chromosomal abnormalities were evenly distributed. Among them, SGA newborns with chromosomal abnormalities were more likely to have poor prognosis. For SGA newborns without a genetic diagnosis, potential causative genes for SGA and SGA with poor prognosis were identified through rare-variant collapsing analysis. Our novel SGA prognosis prediction model, which integrated both genetic and clinical factors, outperformed models relying merely on clinical factors. Overall, the application of NGS in hospitalized SGA newborns shows promise in early genetic diagnosis and prognosis prediction.

### Supplementary Information

**Additional file 1: Additional method.** The inclusion criteria of the China Neonatal Genomes Project (CNGP). **Table S1.** Uniparental disomy (UPD) types and disease associated with growth failure. **Table S2.** The matching results of cases and controls for gestational age, sex, and whether the pregnant mother had gestational hypertension by matching (PSM). **Table S3.** Characteristics of SGA newborns with different genetic diagnosis. **Table S4.** The OMIM diseases and detailed developmental phenotypes for SGA-related genes. **Table S5.** The OMIM diseases and the relevance of disease to underdevelopmental abnormalities for non SGA-related genes. **Table S6.** The OMIM diseases and multiple organs or systems involved for syndromic genes. **Table S7.** Monogenetic variants result. **Table S8.** Characteristics of SGA newborns with different monogenic variants results. **Table S9.** Chromosomal abnormalities result. **Table S10.** The risk genes identified based on the gene burden test. **Table S11.** The baseline information of the SGA-model generation dataset and the SGA-validation dataset.

**Additional file 2: Figure S1.** Flowchart of SGA prognosis prediction model.

## Data Availability

The datasets supporting the major results/conclusions of this article are listed within the article and its additional files. The variation data reported in this paper has been deposited in the Genome Variation Map in National Genomics Data Center, China National Center for Bioinformation / Beijing Institute of Genomics, Chinese Academy of Sciences, under accession number GVM000599 that can be publicly accessible at https://ngdc.cncb.ac.cn/gvm/getProjectDetail?project=GVM000599 [45]. The code for the gene burden test method and the prediction model generation and validation used in this study has been deposited in GitHub and can be publicly accessible at https://github.com/chenhy-lab/SGA_burden.

## Abbreviations

AGA: Appropriate for gestational age
AUC: Area under the receiver operating characteristic curve
CES: Clinical exome sequencing
CNGP: China Neonatal Genomes Project
CNV: Copy number variation
FDR: False discovery rate
GBM: Gradient Boosting Machine
LOH: Loss of heterozygosity
MIS: Missense or non-synonymous variant
MS-MLPA: Methylation-Specific Multiplex ligation-dependent probe amplification
NICU: Newborn intensive care unit
NGS: Next-generation sequencing
NON: Non-coding variant
PSM: Propensity score matching
PTV: Protein-truncating variant
PWS: Prader–Willy syndrome
SGA: Small for gestational age
SNV: Single-nucleotide variation
SYN: Synonymous variant
UPD: Uniparental disomy
