# Supplementary material for: Comprehensive assessment of the genetic characteristics of small for gestational age newborns in NICU: from diagnosis of genetic disorders to prediction of prognosis
Source: Genome Med. 2023 Dec 13;15:112. doi: 10.1186/s13073-023-01268-2 (PMC10717355; doi:10.1186/s13073-023-01268-2)
Supplement: Supplementary file 1 — Additional file 1: Additional method. The inclusion criteria of the China Neonatal Genomes Project (CNGP). Table S1. Uniparental disomy (UPD) types and disease associated with growth failure. Table S2. The matching results of cases and controls for gestational age, sex, and whether the pregnant mother had gestational hypertension by matching (PSM). Table S3. Characteristics of SGA newborns with different genetic diagnosis. Table S4. The OMIM diseases and detailed developmental phenotypes for SGA-related genes. Table S5. The OMIM diseases and the relevance of disease to underdevelopmental abnormalities for non SGA-related genes. Table S6. The OMIM diseases and multiple organs or systems involved for syndromic genes. Table S7. Monogenetic variants result. Table S8. Characteristics of SGA newborns with different monogenic variants results. Table S9. Chromosomal abnormalities result. Table S10. The risk genes identified based on the gene burden test. Table S11. The baseline information of the SGA-model generation dataset and the SGA-validation dataset. [file 13073_2023_1268_MOESM1_ESM.docx]

**Additional file**

[**Additional method. 2**](#_Toc152407279)

[**Table S1. Uniparental disomy (UPD) types and disease associated with growth failure. 3**](#_Toc152407280)

[**Table S2. The matching results of cases and controls for gestational age, sex, and whether the pregnant mother had gestational hypertension by matching (PSM). 4**](#_Toc152407281)

[**Table S3. Characteristics of SGA newborns with different genetic diagnosis. 5**](#_Toc152407282)

[**Table S4. The OMIM diseases and detailed developmental phenotypes for SGA-related genes. 6**](#_Toc152407283)

[**Table S5. The OMIM diseases and the relevance of disease to underdevelopmental abnormalities for non SGA-related genes. 7**](#_Toc152407284)

[**Table S6. The OMIM diseases and multiple organs or systems involved for syndromic genes. 9**](#_Toc152407285)

[**Table S7. Monogenetic variants result. 10**](#_Toc152407286)

[**Table S8. Characteristics of SGA newborns with different monogenic variants results. 14**](#_Toc152407287)

[**Table S9. Chromosomal abnormalities result. 15**](#_Toc152407288)

[**Table S10. The risk genes identified based on the gene burden test. 17**](#_Toc152407289)

[**Table S11. The baseline information of the SGA-model generation dataset and the SGA-validation dataset. 22**](#_Toc152407290)

Additional method.

**The inclusion criteria of the China Neonatal Genomes Project (CNGP)**

Patients evaluated in this study were recruited through the China Neonatal Genomes Project (CNGP), primarily from NICU of Children's Hospital of Fudan University, Shanghai, China. This study was approved by the ethics committees of Children's Hospital of Fudan University (2015-169). All samples used in this study were collected with written informed consent by patients' parents or legal guardians.

The patients included in the CNGP were those who were suspected of having a genetic disorder, and patients older than 1 month at the time of hospital admission were excluded from the CNGP. The inclusion criteria of the CNGP have been previously published[1]. Eligible patients had one or more of the following clinical conditions:

(1) Craniofacial deformities;

(2) central nervous system anomalies including cerebral dysplasia, hydrocephalus, seizure/epilepsy, hypotonia/ hypertonia, intracranial hemorrhage, and central nervous system infection;

(3) cardiovascular abnormalities including congenital heart defect, cardiomyopathy, arrhythmia, and cardiac insufficiency;

(4) evidence of metabolic disease including patients suspected of an inborn error of metabolism by Tandem mass spectrometry analyses, hyperglycemia/hypoglycemia, severe electrolyte disturbance, metabolic acidosis, hyperammonemia, and thyroid dysfunction;

(5) digestive system anomalies including deformity of digestive system, abdominal fissure/hernia, feeding problem, hepatosplenomegaly, cholestasis, recurrent diarrhea;

(6) respiratory system anomalies including choanal atresia, laryngomalacia, pulmonary hypoplasia, pneumothorax, hydrothorax, congenital central hypoventilation syndrome, severe respiratory failure;

(7) skeletal abnormality including skeletal dysplasia, joint deformity, digit malformation, and fracture;

(8) urinary or reproductive system abnormalities including renal dysplasia, ureter abnormality, hypospadias, cryptorchidism, or renal insufficiency;

(9) infection and immune involvement including cellular or humoral immunodeficiency, sepsis;

(10) hematologic abnormalities including coagulation abnormalities, anemia/polycythemia, thrombocytopenia, disseminated intravascular coagulation, hemophagocytic syndrome.

The congenital malformation included cerebral abnormalities, congenital heart disease, digestive tract malformations, skeletal malformation, and special face feature.

**Reference**

1. Xiao F, Yan K, Wang H, Wu B, Hu L, Yang L et al: **Protocol of the China Neonatal Genomes Project: An observational study about genetic testing on 100,000 neonates**. *Pediatric Medicine* 2021, **4**:1-5.

Table S1. Uniparental disomy (UPD) types and disease associated with growth failure.

| **UPD types** | **Chromosome region** | **Disease** | **OMIM** |
| --- | --- | --- | --- |
| UPD(7)mat | 7p12-p14 | Silver-Russell syndrome, SRS | #618905 |
| UPD(14)mat | 14q32 | Temple syndrome, TS | #616222 |
| UPD(15)mat | 15q11.2 | Prader-Willi syndrome, PWS | #176270 |
| UPD(20)mat | 20q11-q13 | Mulchandani-Bhoj-Conlin syndrome, MBCS | #617352 |
| UPD(6)pat | 6q24 | Transient neonatal diabetes mellitus,TNDM | #601410 |

Table S2. The matching results of cases and controls for gestational age, sex, and whether the pregnant mother had gestational hypertension by matching (PSM).

The matching results of SGA (case, n=627) vs. AGA (control, n=1317)

|  | Overall (n = 1944) | case (n = 627) | control (n = 1317) | P value |
| --- | --- | --- | --- | --- |
| Gestational age, mean (SD), week | 35.897 (3.360) | 35.831 (3.357) | 35.928 (3.362) | 0.5 |
| Birth weight, mean (SD), g | 2,426.72 (780.70) | 1,954.02 (634.15) | 2,651.76 (742.60) | <0.001 |
| Gestational age classification (%) | | | | 0.8 |
| Extreme preterm | 10 (0.5) | 3 (0.5) | 7 (0.5) |  |
| Very preterm | 235 (12.1) | 74 (11.8) | 161 (12.2) |  |
| Moderate preterm | 220 (11) | 74 (11.8) | 146 (11.1) |  |
| Late preterm | 504 (25.9) | 174 (27.8) | 330 (25.1) |  |
| Term | 896 (46.1) | 276 (44.0) | 620 (47.6) |  |
| Post term | 79 (4.1) | 26 (4.1) | 53 (4.0) |  |
| Sex (%) | | | | 0.15 |
| Male | 1139 (58.6) | 382 (60.9) | 757 (57.5) |  |
| Female | 805 (41.4) | 245 (39.1) | 560 (42.5) |  |
| The pregnant mother with gestational hypertension (%) | | | | 0.34 |
| Yes | 467 (24.0) | 159 (25.4) | 308 (23.4) |  |
| No | 1477 (76.0) | 468 (74.6) | 1009 (76.6) |  |

The matching results of SGA with poor prognosis (case, n=88) vs. SGA without poor prognosis (control, n=312)

|  | Overall (n = 400) | case (n = 88) | control (n = 312) | P value |
| --- | --- | --- | --- | --- |
| Gestational age, mean (SD), week | 35.89 (3.39) | 35.53 (3.74) | 35.99 (3.28) | 0.43 |
| Birth weight, mean (SD), g | 1,976.33 (642.63) | 1,910.45 (701.82) | 1,994.90 (624.87) | 0.37 |
| Gestational age classification (%) | | | | 0.18 |
| Extreme preterm | 2 (0.5) | 2 (2.3) | 0 (0) |  |
| Very preterm | 45 (11.3) | 13 (14.8) | 32 (10.3) |  |
| Moderate preterm | 47 (11.8) | 10 (11.4) | 37 (11.9) |  |
| Late preterm | 118 (29.5) | 22 (25.0) | 96 (30.8) |  |
| Term | 168 (42.0) | 37 (42.0) | 131 (42.0) |  |
| Post term | 20 (5.0) | 4 (4.5) | 16 (5.1) |  |
| Sex (%) | | | | 0.92 |
| Male | 271 (67.8) | 60 (68.2) | 211 (67.6) |  |
| Female | 129 (32.3) | 28 (31.8) | 101 (32.4) |  |
| The pregnant mother with gestational hypertension (%) | | | | 0.44 |
| Yes | 88 (22.0) | 22 (25.0) | 66 (21.2) |  |
| No | 312 (78.0) | 66 (75.0) | 246 (78.8) |  |

Table S3. Characteristics of SGA newborns with different genetic diagnosis.

|  | **SGA newborns with chromosomal abnormalities (n = 46)** | **SGA newborns with monogenic diseases (n = 42)** | **P value** * |
| --- | --- | --- | --- |
| **Severe SGA (%)** | 28 (60.1) | 20 (47.6) | 0.28 |
| **Death (%)** | 8 (17.4) | 9 (21.4) | 0.79 |
| **Developmental delay (%)** | 25 (54.3) | 14 (33.3) | 0.056 |
| **Growth delay (%)** | 21 (45.7) | 8 (19.0) | 0.012 |
| **Neurodevelopmental delay (%)** | 21 (45.7) | 8 (19.0) | 0.012 |
| **Presenting both physical growth delay and neurodevelopmental delay (%)** | 17 (37.0) | 2 (4.8) | 0.00021 |

* P values are from Fisher’s exact test.

Table S4. The OMIM diseases and detailed developmental phenotypes for SGA-related genes.

| SGA-related genes | Diseases | Phenotypes associated with developmental abnormalities |
| --- | --- | --- |
| *ANKRD11* | KBG syndrome, [MIM:148050] | Clinical features in OMIM contains phenotypes associated with developmental abnormalities: Short stature, microcephaly, delayed bone maturation, developmental delay, etc. |
| *CHD7* | CHARGE syndrome, [MIM:214800]; Hypogonadotropic hypogonadism 5 with or without anosmia, [MIM:612370] | Clinical features in OMIM contains a wide range of congenital malformations and contains phenotypes associated with developmental abnormalities: multiple congenital anomalies, mental retardation, etc. |
| *CREBBP* | Rubinstein-Taybi syndrome 1, [MIM:180849]; Menke-Hennekam syndrome 1, [MIM:618332] | Clinical features in OMIM contains phenotypes associated with developmental abnormalities: Short stature, microcephaly, mental retardation, etc. |
| *EP300* | Menke-Hennekam syndrome 2, [MIM:618333]; Rubinstein-Taybi syndrome 2, [MIM:613684] | Clinical features in OMIM contains phenotypes associated with developmental abnormalities: multiple congenital anomalies, microcephaly, mental retardation, etc. |
| *INSR* | Diabetes mellitus, insulin-resistant, with acanthosis nigricans, [MIM:610549]; Leprechaunism, [MIM:246200]; Rabson-Mendenhall syndrome, [MIM:262190] | Clinical features in OMIM contains phenotypes associated with developmental abnormalities: Short stature, weight less than 5th percentile, small for gestational age, etc. |
| *JAG1* | Alagille syndrome 1, [MIM:118450]; Tetralogy of Fallot, [MIM:187500] | Clinical features in OMIM contains phenotypes associated with developmental abnormalities: multiple congenital anomalies, mental retardation, etc. |
| *NIPBL* | Cornelia de Lange syndrome 1, [MIM:122470] | Clinical features in OMIM contains phenotypes associated with developmental abnormalities: Prenatal growth retardation, short stature, mental retardation, etc. |
| *PEX7* | Peroxisome biogenesis disorder 9B, [MIM:614879]; Rhizomelic chondrodysplasia punctata, type 1, [MIM:215100] | Clinical features in OMIM contains phenotypes associated with developmental abnormalities:bone dysplasia and developmental delay |
| *PRMT7* | Short stature, brachydactyly, intellectual developmental disability, and seizures, [MIM:617157] | Clinical features in OMIM contains phenotypes associated with developmental abnormalities: Short stature, intellectual developmental disability, etc. |
| *PTDSS1* | Lenz-Majewski hyperostotic dwarfism, [MIM:151050] | Clinical features in OMIM contains phenotypes associated with developmental abnormalities: Failure to thrive, intrauterine growth retardation, mental retardation, etc. |
| *TREX1* | Vasculopathy, retinal, with cerebral leukoencephalopathy and systemic manifestations, [MIM:192315]; Aicardi-Goutieres syndrome 1, dominant and recessive, [MIM:225750]; Chilblain lupus, [MIM:610448] | Clinical features in OMIM contains phenotypes associated with developmental abnormalities: microcephaly, developmental retardation, etc. |
| *ZEB2* | Mowat-Wilson syndrome, [MIM:235730] | Clinical features in OMIM contains phenotypes associated with developmental abnormalities: Short stature, microcephaly,mental retardation, etc. |

Table S5. The OMIM diseases and the relevance of disease to underdevelopmental abnormalities for non SGA-related genes.

| Gene | Disease | Disease phenotype | Phenotypes that possibly associated with underdevelopmental abnormalities |
| --- | --- | --- | --- |
| *ABCC8* | Diabetes mellitus, noninsulin-dependent [125853]; Diabetes mellitus, permanent neonatal 3, with or without neurologic features [618857]; Diabetes mellitus, transient neonatal 2 [610374]; et al. | Endocrine and metabolic | Not reported |
| *ACTA1* | Myopathy, actin, congenital, with cores [161800]; Nemaline myopathy 3, autosomal dominant or recessive [161800]; Myopathy, congenital, with fiber-type disproportion 1 [255310]; et al. | Musculoskeletal | Delayed motor development |
| *ANK1* | Spherocytosis, type 1 [182900] | Hematologic | Not reported |
| *COL7A1* | EBD inversa [226600]; Epidermolysis bullosa dystrophica, AR [226600]; Toenail dystrophy, isolated [607523]; et al. | Skin and hair | Not reported |
| *F8* | Hemophilia A [306700] | Hematologic | Not reported |
| *G6PD* | Hemolytic anemia, G6PD deficient (favism) [300908] | Endocrine and metabolic | Not reported |
| *GBA* | Gaucher disease, perinatal lethal [608013]; Gaucher disease, type I [230800]; Gaucher disease, type II [230900]; et al. | Endocrine and metabolic | Postnatal progressive physical growth delay and neurodevelopmental delay |
| *GCDH* | Glutaricaciduria, type I [231670] | Endocrine and metabolic | Failure to thrive |
| *KCNJ11* | Diabetes mellitus, transient neonatal 3 [610582]; Diabetes, permanent neonatal 2, with or without neurologic features [618856]; Hyperinsulinemic hypoglycemia, familial, 2 [601820]; et al. | Endocrine and metabolic | Not reported |
| *KCNQ2* | Seizures, benign neonatal, 1 [121200]; Developmental and epileptic encephalopathy 7 [613720]; Myokymia [121200] | Neurologic | Not reported |
| *LAMA3* | Epidermolysis bullosa, junctional, Herlitz type [226700]; Laryngoonychocutaneous syndrome [245660]; Epidermolysis bullosa, generalized atrophic benign [226650] | Skin and hair | Not reported |
| *LAMB3* | Amelogenesis imperfecta, type IA [104530]; Epidermolysis bullosa, junctional, Herlitz type [MIM226700]; Epidermolysis bullosa, junctional, non-Herlitz type [MIM226650] | Skin and hair | Not reported |
| *MMUT* | Methylmalonic aciduria, mut(0) type [251000] | Endocrine and metabolic | Failure to thrive |
| *MTM1* | Myotubular myopathy, X-linked [310400] | Musculoskeletal | Not reported |
| *NOTCH1* | Adams-Oliver syndrome 5 [616028]; Aortic valve disease 1 [109730] | Syndromic | Terminal transverse limb defects and congenital heart defects |
| *PCCA* | Propionicacidemia [606054] | Endocrine and metabolic | Postnatal short stature |
| *PHOX2B* | Central hypoventilation syndrome, congenital, 1, with or without Hirschsprung disease [209880] | Syndromic | Not reported |
| *RPS19* | Diamond-Blackfan anemia 1 [105650] | Hematologic | Not reported |
| *SERAC1* | 3-methylglutaconic aciduria with deafness, encephalopathy, and Leigh-like syndrome [614739] | Endocrine and metabolic | Failure to thrive |
| *TAF2* | Mental retardation, autosomal recessive 40 [615599] | Neurologic | Postnatal developed microcephaly |

Table S6. The OMIM diseases and multiple organs or systems involved for syndromic genes.

| Gene | Diseases | Multiple organs or systems involved in the syndrome |
| --- | --- | --- |
| *ANKRD11* | KBG syndrome, [MIM:148050] | Head and neck, skeletal system, neurologic system, genitourinary system, skin and hair |
| *CHD7* | Hypogonadotropic hypogonadism 5 with or without anosmia, [MIM:612370]; CHARGE syndrome, [MIM:214800] | Head and neck, gastrointestinal system, cardiovascular system, skeletal system, neurologic system, genitourinary system, immune system |
| *CREBBP* | Rubinstein-Taybi syndrome 1, [MIM:180849]; Menke-Hennekam syndrome 1, [MIM:618332] | Head and neck, cardiovascular system, skeletal system, neurologic system, genitourinary system, immune system |
| *EP300* | Menke-Hennekam syndrome 2,[MIM:618333]; Rubinstein-Taybi syndrome 2,[MIM:613684] | Head and neck, skeletal system, neurologic system, gastrointestinal system |
| *INSR* | Diabetes mellitus, insulin-resistant, with acanthosis nigricans, [MIM:610549]; Leprechaunism, [MIM:246200]; Rabson-Mendenhall syndrome, [MIM:262190] | Endocrine system, head and neck, neurologic system, genitourinary system, skin and hair |
| *JAG1* | Alagille syndrome 1, [MIM:118450]; Tetralogy of Fallot, [MIM:187500] | Gastrointestinal system, cardiovascular system, skeletal system, head and neck, genitourinary system |
| *NIPBL* | Cornelia de Lange syndrome 1, [MIM:122470] | Head and neck, skeletal system, neurologic system, genitourinary system |
| *NOTCH1* | Adams-Oliver syndrome 5,[MIM:616028]; Aortic valve disease 1,[MIM:109730] | Head and neck, cardiovascular system, skeletal system, neurologic system, skin and hair |
| *PHOX2B* | Central hypoventilation syndrome, congenital, 1, with or without Hirschsprung disease,[MIM:209880] | Respiratory system, gastrointestinal system, neurologic system |
| *PRMT7* | Short stature, brachydactyly, intellectual developmental disability, and seizures,[MIM:617157] | Head and neck, skeletal system, neurologic system |
| *TREX1* | Vasculopathy, retinal, with cerebral leukoencephalopathy and systemic manifestations, [MIM:192315]; Aicardi-Goutieres syndrome 1, dominant and recessive, [MIM:225750]; Chilblain lupus, [MIM:610448] | Head and neck, neurologic system |
| *ZEB2* | Mowat-Wilson syndrome, [MIM:235730] | Head and neck, cardiovascular system, neurologic system, gastrointestinal system |

Table S7. Monogenetic variants result.

| Patient Number | Clinical phenotypes / diagnosis | Gene | Genomic reference sequence | Transcript | Nucleotide change | Amino acid change | Zygosite | Variant type | gnomAD | Disease  [OMIM number] | Gene classification | Disease phenotype | Inheritance pattern | In silico tools | HGMD included (PMID) | Sanger | Evidence | Pathogenicity |
| --- | --- | --- | --- | --- | --- | --- | --- | --- | --- | --- | --- | --- | --- | --- | --- | --- | --- | --- |
| 4 | Lenz-Majewski syndrome | *PTDSS1* | chr8: g.97316363C>T | NM_014754.3 | c.848C>T | p.Ser283Phe | Het | missense | 0 | Lenz-Majewski hyperostotic dwarfism [151050] | SGA-related | Musculoskeletal | AD | BayesDel: PP3_Supporting (0.19) | 25741868 | *De novo* | PS2, PM1, PP3_Moderate, PM2 | LP |
| 14 | CHARGE syndrome | *CHD7* | chr8: g.61693846del | NM_017780.4 | c.1953del | p.Asp652ThrfsTer59 | Het | stop gained | 0 | Hypogonadotropic hypogonadism 5 with or without anosmia [612370]; CHARGE syndrome [214800] | SGA-related | Syndromic | AD | - | - | *De novo* | PVS1_VeryStrong, PS2, PM2 | P |
| 20 | 3-methylglutaconic aciduria | *SERAC1* | chr6: g.158567859G>A | NM_032861.4 | c.442C>T | p.Arg148Ter | Hom | stop gained | 0 | 3-methylglutaconic aciduria with deafness, encephalopathy, and Leigh-like syndrome [614739] | non SGA-related | Endocrine and metabolic | AR | BayesDel: PP3_Strong (0.66) | 22683713 | NA | PVS1_VeryStrong, PP3_Strong, PM3, PM2 | P |
| 21 | Seizures | *KCNQ2* | chr20: g.62073758C>T | NM_001382235.1 | c.816+1G>A |  | Het | splicing | 0 | Seizures, benign neonatal, 1 [121200]; Developmental and epileptic encephalopathy 7 [613720]; Myokymia [121200] | non SGA-related | Neurologic | AD | BayesDel: PP3_Strong (0.66) | - | NA | PVS1_VeryStrong, PM2 | LP |
| 30 | Alagille syndrome | *JAG1* | chr20: g.10625625G>A | NM_000214.3 | c.2230C>T | p.Arg744Ter | Het | stop gained | 0 | Alagille syndrome 1 [118450]; Tetralogy of Fallot [187500] | SGA-related | Syndromic | AD | BayesDel: PP3_Strong (0.54) | 9585603 | NA | PVS1_VeryStrong, PP3_Strong, PM2 | P |
| 51 | Seizures | *KCNQ2* | chr20: g.62046439G>A | NM_172107.4 | c.1342C>T | p.Arg448Ter | Het | stop gained | 0 | Seizures, benign neonatal, 1 [121200]; Developmental and epileptic encephalopathy 7 [613720]; Myokymia [121200] | non SGA-related | Neurologic | AD | BayesDel: PP3_Strong (0.66) | 11690625 | NA | PVS1_VeryStrong, PP3_Strong, PM2 | P |
| 54 | Neonatal diabetes mellitus | *ABCC8* | chr11: g.17436118C>T | NM_000352.6 | c.2331G>A | p.Trp777Ter | Het | stop gained | 0 | Diabetes mellitus, noninsulin-dependent [125853]; Diabetes mellitus, permanent neonatal 3, with or without neurologic features [618857]; Diabetes mellitus, transient neonatal 2 [610374]; et al. | non SGA-related | Endocrine and metabolic | AD | BayesDel: PP3_Strong (0.66) | 30352420 | Paternal | PVS1_VeryStrong, PM2 | LP |
| 66 | Rabson-Mendenhall Syndrome | *INSR* | chr19: g.7122904G>A | NM_000208.4 | c.3355C>T | p.Arg1119Trp | Het | missense | 0 | Diabetes mellitus, insulin-resistant, with acanthosis nigricans [610549]; Leprechaunism [246200]; Rabson-Mendenhall syndrome [262190] | SGA-related | Syndromic | AD/AR | BayesDel: PP3 _Moderate (0.41) | 29082893 | Maternal | PP3_Moderate, PM1, PM2, PP4 | LP |
|  |  |  | chr19: g.7150507_ 7152938del | - | 2.43Kb deletion |  | Het | microdeletion | 0 |  |  |  | AD/AR | - | 29082893 | Paternal | - | P |
| 73 | Aicardi-Goutieres syndrome | *TREX1* | chr3: g.48508348dup | NM_033629.6 | c.294dup | p.Cys99MetfsTer3 | Hom | frameshift | 0.0001 | Vasculopathy, retinal, with cerebral leukoencephalopathy and systemic manifestations [192315]; Aicardi-Goutieres syndrome 1, dominant and recessive [225750]; Chilblain lupus [610448] | SGA-related | Syndromic | AR | - | 24183309 | NA | PVS1_VeryStrong, PM2 | LP |
| 83 | Mental retardation | *TAF2* | chr8: g.120793333_ 120793336del | NM_003184.4 | c.2216_2219del | p.Asn739ThrfsTer3 | Het | frameshift | 0 | Mental retardation, autosomal recessive 40 [615599] | non SGA-related | Neurologic | AR | - | - | Maternal | PVS1_VeryStrong, PM2 | LP |
|  |  |  | chr8: g.120793427T>C | NM_003184.4 | c.2119A>G | p.Asn707Asp | Het | missense | 3.229e-05 |  |  |  | AR | BayesDel: BP4_Supporting (-0.31) | - | Paternal | PM3_Strong, PM2, PP2 | LP |
| 87 | CHARGE syndrome | *CHD7* | chr8: g.61728951_ 61728955del | NM_017780.4 | c.2504_2508del | p.Tyr835SerfsTer14 | Het | frameshift | 0 | Hypogonadotropic hypogonadism 5 with or without anosmia [612370]; CHARGE syndrome [214800] | SGA-related | Syndromic | AD | - | - | NA | PVS1_VeryStrong, PM2 | LP |
| 103 | Methylmalonic aciduria | *MMUT* | chr6: g.49419405C>T | NM_000255.4 | c.1106G>A | p.Arg369His | Het | missense | 3.236e-05 | Methylmalonic aciduria, mut(0) type [251000] | non SGA-related | Endocrine and metabolic | AR | BayesDel: PP3_Strong (0.59) | 9929975 | NA | PM3_Strong, PP3_Strong, PM1, PM2 | P |
|  |  |  | chr6: g.49421467A>G | NM_000255.4 | c.914T>C | p.Leu305Ser | Het | missense | 0 |  |  |  | AR | BayesDel: PP3_Strong (0.61) | 16281286 | NA | PM3_Strong, PP3_Strong, PM1, PM2 | P |
| 122 | Rubinstein-Taybi syndrome | *CREBBP* | chr16: g.3786105T>A | NM_004380.3 | c.4660A>T | p.Lys1554Ter | Het | stop gained | 0 | Menke-Hennekam syndrome 1[618332]; Rubinstein-Taybi syndrome 1[180849] | SGA-related | Syndromic | AD | BayesDel: PP3_Strong (0.66) | - | *De novo* | PVS1_VeryStrong, PS2, PP3_Strong, PM2 | P |
| 123 | Seizures | *KCNQ2* | chr20: g.62076065G>A | NM_001382235.1 | c.637C>T | p.Arg213Trp | Het | missense | 0 | Seizures, benign neonatal, 1 [121200]; Developmental and epileptic encephalopathy 7 [613720]; Myokymia [121200] | non SGA-related | Neurologic | AD | BayesDel: PP3_Strong (0.58) | 18353052 | *De novo* | PS2, PP3_Strong, PM1, PM2 | P |
| 153 | Congenital myopathy | *ACTA1* | chr1: g.229567881A>G | NM_001100.4 | c.668T>C | p.Leu223Pro | Het | missense | 0 | Myopathy, actin, congenital, with cores [161800]; Nemaline myopathy 3, autosomal dominant or recessive [161800]; Myopathy, congenital, with fiber-type disproportion 1 [255310]; et al. | non SGA-related | Musculoskeletal | AD | BayesDel: PP3_Moderate (0.49) | 15468086 | *De novo* | PS2, PP3_Strong, PM2, PP2 | P |
| 156 | Epidermolysis bullosa | *LAMA3* | chr18: g.21494502del | NM_198129.4 | c.7458del | p.Met2487TrpfsTer4 | Het | frameshift | 0 | Epidermolysis bullosa, junctional, Herlitz type [226700]; Laryngoonychocutaneous syndrome [245660]; Epidermolysis bullosa, generalized atrophic benign [226650] | non SGA-related | Skin and hair | AR | - | - | Maternal | PVS1_VeryStrong, PM3, PM2 | P |
|  |  |  | chr18: g.21508201G>A | NM_198129.4 | c.8292G>A | p.Trp2764Ter | Het | stop gained | 0 |  |  |  | AR | BayesDel: PP3_Strong (0.66) | - | Paternal | PVS1_VeryStrong, PP3_Strong, PM3, PM2 | P |
| 163 | Peroxisome biogenesis disorder | *PEX7* | chr6: g.137146404del | NM_000288.4 | c.183del | p.Phe61LeufsTer13 | Het | frameshift | 3.23e-05 | Peroxisome biogenesis disorder 9B [614879]; Rhizomelic chondrodysplasia punctata, type 1 [215100] | SGA-related | Endocrine and metabolic | AR | - | 12325024 | Maternal | PVS1_VeryStrong, PM3 | LP |
|  |  |  | chr6: g.137147551T>G | NM_000288.4 | c.283T>G | p.Trp95Gly | Het | missense | 0 |  |  |  | AR | BayesDel: PP3_Strong (0.58) | - | Paternal | PP3_Strong, PM1, PM2 | P |
| 174 | G6PD deficiency | *G6PD* | chrX: g.153760472C>T | NM_001360016.2 | c.1388G>A | p.Arg493His | Hemi | missense | 0.0003 | Hemolytic anemia, G6PD deficient (favism) [300908] | non SGA-related | Endocrine and metabolic | XLD | BayesDel: PP3_Strong (0.77) | 1953767 | NA | PS3, PS4, PP4(Clinical diagnosis: G6PD deficiency) | P |
| 177 | Spherocytosis and severe anemia | *ANK1* | chr8: g.41571762dup | NM_001142446.2 | c.1816dup | p.Leu606ProfsTer48 | Het | frameshift | 0 | Spherocytosis, type 1 [182900] | non SGA-related | Hematologic | AD | - | 8640229 | NA | PVS1_VeryStrong, PM2 | LP |
| 182 | Alagille syndrome | *JAG1* | chr20: g.10633139C>T | NM_000214.3 | c.863G>A | p.Trp288Ter | Het | stop gained | 0 | Alagille syndrome 1 [118450]; Tetralogy of Fallot [187500] | SGA-related | Syndromic | AD | BayesDel: PP3_Strong (0.59) | - | Maternal | PVS1_VeryStrong, PP3_Strong, PM2 | P |
| 185 | Epidermolysis bullosa dystrophica | *COL7A1* | chr3: g.48602593C>A | NM_000094.4 | c.8569G>T | p.Glu2857Ter | Het | stop gained | 0 | EBD inversa [226600]; Epidermolysis bullosa dystrophica, AR [226600]; Toenail dystrophy, isolated [607523]; et al. | non SGA-related | Skin and hair | AR | BayesDel: PP3_Strong (0.66) | 8592061 | Paternal | PVS1_VeryStrong, PP3_Strong, PM3, PM2 | P |
|  |  |  | chr3: g.48622187G>A | NM_000094.4 | c.4027C>T | p.Arg1343Ter | Het | stop gained | 6.471e-05 |  |  |  | AR | BayesDel: PP3_Strong (0.66) | 8037207 | Maternal | PVS1_VeryStrong, PP3_Strong, PM3, PM2 | P |
| 264 | Peroxisome biogenesis disorder | *PEX7* | chr6: g.137147551T>G | NM_000288.4 | c.283T>G | p.Trp95Gly | Het | missense | 0 | Peroxisome biogenesis disorder 9B [614879]; Rhizomelic chondrodysplasia punctata, type 1 [215100] | SGA-related | Endocrine and metabolic | AR | BayesDel: PP3_Strong (0.58) | - | Paternal | PP3_Strong, PM1, PM2 | P |
|  |  |  | chr6: g.137146404del | NM_000288.4 | c.183del | p.Phe61LeufsTer13 | Het | frameshift | 3.23e-05 |  |  |  | AR | - | 12325024 | Maternal | PVS1_VeryStrong, PM3 | LP |
| 271 | Epidermolysis bullosa dystrophica | *COL7A1* | chr3: g.48624473C>A | NM_000094.4 | c.3208G>T | p.Glu1070Ter | Het | stop gained | 0 | EBD inversa [226600]; Epidermolysis bullosa dystrophica, AR [226600]; Toenail dystrophy, isolated [607523]; et al. | non SGA-related | Skin and hair | AR | BayesDel: PP3_Strong (0.66) | - | Maternal | PVS1_VeryStrong, PP3_Strong, PM3, PM2 | P |
|  |  |  | chr3: g.48602593C>A | NM_000094.4 | c.8569G>T | p.Glu2857Ter | Het | stop gained | 0 |  |  |  | AR | BayesDel: PP3_Strong (0.66) | 8592061 | Paternal | PVS1_VeryStrong, PP3_Strong, PM3, PM2 | P |
| 294 | Diamond-Blackfan anemia | *RPS19* | chr19: g.42373170del | NM_001321485.2 | c.255del | p.Thr86ArgfsTer65 | Het | frameshift | 0 | Diamond-Blackfan anemia 1 [105650] | non SGA-related | Hematologic | AD |  | - | NA | PVS1_VeryStrong, PM2 | LP |
| 308 | Propionicacidemia | *PCCA* | chr13: g.100764143G>A | NM_000282.4 | c.231+1G>A |  | Het | splicing | 0 | Propionicacidemia [606054] | non SGA-related | Endocrine and metabolic | AR | BayesDel: PP3_Strong (0.66) | - | NA | PVS1_Strong, PP3_Strong, PM2 | P |
|  |  |  | chr13: g.100861713T>A | NM_000282.4 | c.596T>A | p.Val199Asp | Het | missense | 0 |  |  |  | AR | BayesDel: PP3_Strong (0.53) | - | NA | PP3_Strong, PM1, PM2 | LP |
| 365 | Congenital myopathy | *MTM1* | chrX: g.149814309G>A | NM_001376908.1 | c.832G>A | p.Asp278Asn | Hemi | missense | 0 | Myotubular myopathy, X-linked [310400] | non SGA-related | Musculoskeletal | XLR | BayesDel: PP3_Supporting (0.23) | - | Maternal | PP3_Moderate, PM1, PM2, PP1 | LP |
| 377 | Glutaricaciduria | *GCDH* | chr19: g.13008224G>A | NM_000159.4 | c.1064G>A | p.Arg355His | Het | missense | 0 | Glutaricaciduria, type I [231670] | non SGA-related | Endocrine and metabolic | AR | BayesDel: PP3_Strong (0.54) | 9600243 | Maternal | PP3_Strong, PM1, PM2 | LP |
|  |  |  | chr19: g.13008525C>G | NM_000159.4 | c.1091C>G | p.Pro364Arg | Het | missense | 0 |  |  |  | AR | BayesDel: PP3_Strong (0.51) | - | Paternal | PP3_Strong, PM1, PM2 | LP |
| 389 | Central hypoventilation syndrome | *PHOX2B* | chr4: g.41748007_ 41748027dup | NM_003924.4 | c.756_776dup | p.Ala254_Ala260dup | Het | inframe insertion | 0 | Central hypoventilation syndrome, congenital, 1, with or without Hirschsprung disease [209880] | non SGA-related | Syndromic | AD | - | - | NA | PS2, PM4, PM2, PP4(Clinical diagnosis: Central hypoventilation syndrome) | LP |
| 407 | Epidermolysis bullosa | *LAMB3* | chr1: g.209797020G>A | NM_001017402.2 | c.2188C>T | p.Gln730Ter | Het | stop gained | 0 | Amelogenesis imperfecta, type IA [104530]; Epidermolysis bullosa, junctional, Herlitz type [MIM226700]; Epidermolysis bullosa, junctional, non-Herlitz type [MIM226650] | non SGA-related | Skin and hair | AR | BayesDel: PP3_Strong (0.62) | - | Maternal | PVS1_VeryStrong, PP3_Strong, PM2 | P |
|  |  |  | chr1: g.209799264G>A | NM_001017402.2 | c.1705C>T | p.Arg569Ter | Het | stop gained | 0 |  |  |  | AR | BayesDel: PP3_Strong (0.62) | 8824879 | Paternal | PVS1_VeryStrong, PP3_Strong | P |
| 417 | Congenital megacolon, Abdominal distention, and ventricular septal defect | *ZEB2* | chr2: g.145156674del | NM_014795.4 | c.2080del | p.Gln694AsnfsTer22 | Het | frameshift | 0 | Mowat-Wilson syndrome [235730] | SGA-related | Syndromic | AD | - | - | *De novo* | PVS1_VeryStrong, PS2, PM2 | P |
| 427 | KBG syndrome | *ANKRD11* | chr16: g.89347400G>C | NM_001256182.2 | c.5550C>G | p.Tyr1850Ter | Het | stop gained | 0 | KBG syndrome [148050] | SGA-related | Syndromic | AD | BayesDel: PP3_Moderate (0.30) | - | NA | PVS1_VeryStrong, PP3_Moderate, PM2 | P |
| 525 | Rubinstein-Taybi syndrome | *CREBBP* | chr16: g.3801726C>T | NM_004380.3 | c.3779+1G>A |  | Het | splicing | 0 | Rubinstein-Taybi syndrome 1 [180849]; Menke-Hennekam syndrome 1 [618332] | SGA-related | Syndromic | AD | BayesDel: PP3_Strong (0.66) | - | *De novo* | PVS1_VeryStrong, PS2, PP3_Strong, PM2 | P |
| 536 | Cornelia de Lange syndrome 1 | *NIPBL* | chr5: g.37051938G>C | NM_133433.4 | c.7012G>C | p.Ala2338Pro | Het | missense | 0 | Cornelia de Lange syndrome 1 [122470] | SGA-related | Syndromic | AD | BayesDel: PP3_Strong (0.53) | - | *De novo* | PS2, PP3_Strong, PM1, PM2 | P |
| 559 | Rubinstein-Taybi syndrome | *EP300* | chr22: g.41537040A>G | NM_001429.3 | c.1879-12A>G |  | Het | splicing | 0 | Menke-Hennekam syndrome 2 [618333]; Rubinstein-Taybi syndrome 2 [613684] | SGA-related | Syndromic | AD | SpliceAI: Acceptor loss (0.93) | 26486927 | *De novo* | PS2, PP3, PM2 | LP |
| 564 | Hemophilia A | *F8* | chrX: g.154225262C>T | NM_000132.4 | c.374G>A | p.Trp125Ter | Hemi | stop gained | 0 | Hemophilia A [306700] | non SGA-related | Hematologic | XLR | BayesDel: PP3_Strong (0.83) | - | NA | PVS1_VeryStrong, PP3_Strong, PM2, PP4(Clinical diagnosis: Hemophilia A) | P |
| 567 | CHARGE syndrome | *CHD7* | chr8: g.61734660C>G | NM_017780.4 | c.2913C>G | p.Tyr971Ter | Het | stop gained | 0 | Hypogonadotropic hypogonadism 5 with or without anosmia [612370]; CHARGE syndrome [214800] | SGA-related | Syndromic | AD | BayesDel: PP3_Strong (0.66) | - | *De novo* | PVS1_VeryStrong, PS2, PP3_Strong, PM2 | P |
| 598 | Adams-Oliver syndrome | *NOTCH1* | chr9: g.139395003C>T | NM_017617.3 | c.5934+1G>A |  | Het | splicing | 0 | Adams-Oliver syndrome 5 [616028]; Aortic valve disease 1 [109730] | non SGA-related | Syndromic | AD | - | - | Maternal | PVS1_VeryStrong, PM2 | LP |
| 625 | Short stature | *PRMT7* | chr16: g.68387410del | NM_001351143.3 | c.1591delC | p.Arg531GlyfsTer18 | Hom | frameshift | 0 | Short stature, brachydactyly, intellectual developmental disability, and seizures [617157] | SGA-related | Syndromic | AR | - | - | Maternal/Paternal | PVS1_VeryStrong, PM3, PM2 | P |
| 645 | Seizures | *KCNQ2* | chr20: g.62076052G>A | NM_001382235.1 | c.650C>T | p.Thr217Ile | Het | missense | 0 | Seizures, benign neonatal, 1 [121200]; Developmental and epileptic encephalopathy 7 [613720]; Myokymia [121200] | non SGA-related | Neurologic | AD | BayesDel: PP3_Supporting (0.21) | 23621294 | NA | PP3_Strong, PM1, PM5, PM2 | LP |
| 669 | Epidermolysis bullosa dystrophica | *COL7A1* | chr3: g.48621949del | NM_000094.4 | c.4089del | p.Pro1364LeufsTer35 | Het | frameshift | 0 | EBD inversa [226600]; Epidermolysis bullosa dystrophica, AR [226600]; Toenail dystrophy, isolated [607523]; et al. | non SGA-related | Skin and hair | AD | - | - | NA | PVS1_VeryStrong, PM2 | LP |
| 720 | Gaucher disease | *GBA* | chr1: g.155205518C>G | NM_001005741.3 | c.1342G>C | p.Asp448His | Het | missense | 0.0003 | Gaucher disease, perinatal lethal [608013]; Gaucher disease, type I [230800]; Gaucher disease, type II [230900]; et al. | non SGA-related | Endocrine and metabolic | AR | BayesDel: PP3_Moderate (0.40) | 2269438 | Paternal | PP3_Moderate, PM1, PM3 | LP |
|  |  |  | chr1: g.155207371T>A | NM_001005741.3 | c.762-2A>T |  | Het | splicing | 0 |  |  |  | AR | BayesDel: PP3_Strong (0.66) | - | Maternal | PVS1_VeryStrong, PP3_Strong, PM2 | P |
| 721 | Neonatal diabetes mellitus | *KCNJ11* | chr11: g.17409038G>A | NM_000525.4 | c.601C>T | p.Arg201Cys | Het | missense | 0 | Diabetes mellitus, transient neonatal 3 [610582]; Diabetes, permanent neonatal 2, with or without neurologic features [618856]; Hyperinsulinemic hypoglycemia, familial, 2 [601820]; et al. | non SGA-related | Endocrine and metabolic | AD | BayesDel: PP3_Strong (0.57) | 15115830 | *De novo* | PS2, PP3_Strong, PM1, PM2 | P |

Abbreviations: AD: Autosomal dominant; AR: Autosomal recessive; XLR: X-linked recessive; NA: Not Available; P: pathogenic; LP: likely pathogenic.

Table S8. Characteristics of SGA newborns with different monogenic variants results.

|  | **SGA newborns identified with SGA-related genes(n=17)** | **SGA newborns identified with non-SGA-related genes (n=25)** | **P value*** |
| --- | --- | --- | --- |
| **Severe SGA (%)** | 12 (70.6) | 8 (32.0) | 0.027 |
| **Death (%)** | 6 (35.3) | 3 (12.0) | 0.12 |
| **Developmental delay (%)** | 7 (41.2) | 7 (28.0) | 0.51 |
| **Growth delay (%)** | 6 (35.3) | 2 (8.0) | 0.045 |
| **Neurodevelopmental delay (%)** | 3 (17.6) | 5 (20.0) | >0.99 |
| **Presenting both physical and neurodevelopmental delay (%)** | 2 (11.8) | 0 (0.0) | 0.16 |

* P values are from Fisher’s exact test.

Table S9. Chromosomal abnormalities result.

| **Patient number** | **Clinical phenotypes or diagnosis** | **Chromosomal abnormalities type** | **Chromosomal band suspected to be affected** | **Genomic position of CNV based on CES** | **Size of CNV based on CES** |
| --- | --- | --- | --- | --- | --- |
| 2 | DiGeorge syndrome | Deletion | 22q11.21 | chr22:18900289-21245502 | 2345.213Kb |
| 12 | Prader–Willy syndrome | Deletion | 15q11.2-15q13.1 | chr15:25219457-28327041 | 3107.584Kb |
| 26 | DiGeorge syndrome | Deletion | 22q11.21-22q11.23 | chr22:22123328-24237410 | 2114.082Kb |
| 34 | Klinefelter Syndrome | Karyotype abnormality | X chromosome Duplication | chrX:1-155270560 | 156Mb |
| 36 | Neurodevelopmental delay | Deletion | 13q33.3-13q34 | chr13:108859786-114440708 | 5580.922Kb |
|  |  | Duplication | 1q43-1q44 | chr1:243419342-244220778 | 3281.964Kb |
| 41 | Trisomy 18 syndrome | Karyotype abnormality | Trisomy 18 | chr18:1-78077248 | 80Mb |
| 46 | Congenital heart disease | Duplication | 21q21.1-21q22.3 | chr21:19641432-47865682 | 28224.25Kb |
| 47 | Prader–Willy syndrome | Deletion | 15q11.2-15q13.1 | chr15:23043278-28327041 | 5283.763Kb |
| 50 | Trisomy 18 syndrome | Karyotype abnormality | Trisomy 18 | chr18:1-78077248 | 80Mb |
| 111 | Prader–Willy syndrome | Deletion | 15q11.2-15q13.1 | chr15:25219457-28327041 | 3107.584Kb |
| 112 | DiGeorge syndrome | Deletion | 22q11.21 | chr22:18900289-21245502 | 2345.213Kb |
| 116 | Congenital heart disease and neurodevelopmental delay | Deletion | 1p36.33-1p36.32 | chr1:955499-2344003 | 1388.504Kb |
| 160 | Trisomy 18 syndrome | Karyotype abnormality | Trisomy 18 | chr18:1-78077248 | 80Mb |
| 179 | Trisomy 18 syndrome | Karyotype abnormality | Trisomy 18 | chr18:1-78077248 | 80Mb |
| 181 | Multiple malformations | Deletion | 15q26.3 | chr15:99191767-101792253 | 2600.486Kb |
|  |  | Duplication | 2q36.3-2q37.3 | chr2:227867429-242708226 | 14840.797Kb |
| 195 | Prader–Willy syndrome | Deletion | 15q11.2-15q13.1 | chr15:23888695-28327041 | 4438.346Kb |
| 213 | Prader–Willy syndrome | Deletion | 15q11.2-15q13.1 | chr15:23888695-28327041 | 4438.346Kb |
| 216 | Growth delay | Deletion | 7q36.1-7q36.3 | chr7:148504474-157210133 | 8705.659Kb |
| 220 | Prader–Willy syndrome | Deletion | 15q11.2-15q13.1 | chr15:23888695-28327041 | 4438.346Kb |
| 235 | Trisomy 18 syndrome | Karyotype abnormality | Trisomy 18 | chr18:1-78077248 | 80Mb |
| 351 | Lissencephaly, growth delay and neurodevelopmental delay | Deletion | 17p13.3 | chr17:975853-2588909 | 1613.056Kb |
| 353 | Congenital heart disease | Duplication | 8p23.1 | chr8:6266799-11617511 | 5350.712Kb |
| 363 | Cerebral dysplasia | Deletion | 10q26.12-10q26.2 | chr10:122610873-127505094 | 4894.221Kb |
| 396 | Prader–Willy syndrome | Deletion | 15q11.2-15q13.1 | chr15:23888695-28327041 | 4438.346Kb |
| 482 | Williams syndrome | Deletion | 7q11.23 | chr7:73442118-74203048 | 760.93Kb |
| 485 | Multiple malformations | Deletion | 8p23.3-8p22 | chr8:1719097-18258728 | 16539.631Kb |
| 486 | Multiple malformations | Duplication | 6p22.3-6p25.3 | chr6:391751-18149402 | 17757.651Kb |
| 493 | DiGeorge syndrome | Deletion | 22q11.21 | chr22:18900289-21245502 | 2345.213Kb |
| 510 | Williams syndrome | Deletion | 7q11.23 | chr7:73442118-74203048 | 760.93Kb |
| 511 | Williams syndrome | Deletion | 7q11.23 | chr7:73442118-74203048 | 760.93Kb |
| 539 | Prader–Willy syndrome | Deletion | 15q11.2-15q13.1 | chr15:25219457-28327041 | 3107.584Kb |
| 546 | Trisomy 18 syndrome | Karyotype abnormality | Trisomy 18 | chr18:1-78077248 | 80Mb |
| 561 | Prader–Willy syndrome | Deletion | 15q11.2-15q13.1 | chr15:23888695-28327041 | 4438.346Kb |
| 597 | Multiple malformations | Deletion | 13q22.3-13q32.1 | chr13:77566178-95364799 | 17798.621Kb |
| 617 | Multiple malformations | Karyotype abnormality | Trisomy 9 mosaicism | - | - |
| 634 | Multiple malformations | Duplication | 6p22.3-6p25.3 | chr6:394820-18149402 | 17754.582Kb |
| 635 | Trisomy X syndrome | Karyotype abnormality | X chromosome Duplication | chrX:1-155270560 | 156Mb |
| 639 | Congenital heart disease and neurodevelopmental delay | Deletion | 14q32.32-14q32.33 | chr14:103388999-106322323 | 2933.324Kb |
|  |  | Duplication | 8q24.3 | chr8:143822366-146157976 | 2335.61Kb |
| 659 | Facial deformity, agenesis of the corpus callosum | Duplication | Xq27.3-Xq28 | chrX:144329022-154842613 | 10513.591Kb |
| 664 | DiGeorge syndrome | Deletion | 22q11.21 | chr22:18893832-20307583 | 1413.751Kb |
| 666 | Congenital heart disease | Duplication | 17p11.2 | chr17:16842394-20359984 | 3517.59Kb |
| 671 | Cerebral dysplasia | Deletion | 1q43-1q44 | chr1:241660902-249214145 | 7553.243Kb |
| 683 | Growth delay | Deletion | 3q26.33-3q27.2 | chr3:182511291-185655891 | 3144.6Kb |
| 716 | DiGeorge syndrome | Deletion | 22q11.21 | chr22:18893832-20307583 | 1413.751Kb |
| 722 | Prader–Willy syndrome | Imprinting disorder | 15q11-15q13 | - | - |
| 723 | Prader–Willy syndrome | Imprinting disorder | 15q11-15q13 | - | - |

Abbreviations: CNV: copy number variation; CES: Clinical Exome Sequencing.

Table S10. The risk genes identified based on the gene burden test.

| **The risk genes for SGA identified based on the gene burden test in the comparison of SGA (case, n=627) vs. AGA (control, n=1317)** | | | | | | | | | | | | |
| --- | --- | --- | --- | --- | --- | --- | --- | --- | --- | --- | --- | --- |
| **Gene** | **PTV** | | | | **MIS** | | | | **Risk Score** | **Permutation P value for risk score** | **FDR^3^** |  |
|  | **The number of samples in case** | **The number of samples in control** | **P value** | **FDR^1^** | **The number of samples in case** | **The number of samples in control** | **P value** | **FDR^2^** |  |  |  |  |
| *AFP* | 5 | 1 | 0.015 | >0.99 | 11 | 24 | 0.605 | >0.99 | 3.855 | 0.002 | 0.088 |  |
| *ATXN1* | 0 | 2 | >0.99 | >0.99 | 59 | 90 | 0.030 | >0.99 | 1.526 | 0.206 | 0.811 |  |
| *ATXN3* | 28 | 36 | 0.033 | >0.99 | 46 | 71 | 0.058 | >0.99 | 4.192 | 0.011 | 0.246 |  |
| *BLM* | 0 | 1 | >0.99 | >0.99 | 50 | 74 | 0.031 | >0.99 | 1.508 | 0.082 | 0.593 |  |
| *BMPR2* | 0 | 0 | >0.99 | >0.99 | 19 | 16 | 0.005 | >0.99 | 2.271 | 0.004 | 0.124 |  |
| *BRAF* | 0 | 0 | >0.99 | >0.99 | 33 | 41 | 0.016 | >0.99 | 1.800 | 0.015 | 0.291 |  |
| *C1QB* | 0 | 0 | >0.99 | >0.99 | 5 | 1 | 0.015 | >0.99 | 1.819 | 0.011 | 0.246 |  |
| *C6* | 2 | 3 | 0.518 | >0.99 | 27 | 35 | 0.038 | >0.99 | 1.987 | 0.090 | 0.618 |  |
| *C8A* | 0 | 0 | >0.99 | >0.99 | 23 | 20 | 0.003 | >0.99 | 2.539 | 0.001 | 0.043 |  |
| *CC2D2A* | 3 | 4 | 0.404 | >0.99 | 66 | 99 | 0.017 | >0.99 | 2.548 | 0.039 | 0.445 |  |
| *CDAN1* | 3 | 1 | 0.102 | >0.99 | 64 | 98 | 0.025 | >0.99 | 3.582 | 0.004 | 0.137 |  |
| *CLCN4* | 0 | 0 | >0.99 | >0.99 | 5 | 2 | 0.039 | >0.99 | 1.408 | 0.010 | 0.246 |  |
| *CLCN7* | 3 | 0 | 0.033 | >0.99 | 16 | 29 | 0.369 | >0.99 | 3.384 | 0.002 | 0.098 |  |
| *CLMP* | 0 | 0 | >0.99 | >0.99 | 8 | 3 | 0.007 | >0.99 | 2.160 | 0.005 | 0.153 |  |
| *CNGB1* | 4 | 0 | 0.011 | >0.99 | 54 | 132 | 0.858 | >0.99 | 4.004 | <0.001 | 0.017 |  |
| *COG1* | 3 | 0 | 0.033 | >0.99 | 22 | 43 | 0.437 | >0.99 | 3.311 | 0.002 | 0.073 |  |
| *COG4* | 0 | 0 | >0.99 | >0.99 | 26 | 34 | 0.045 | >0.99 | 1.351 | 0.233 | 0.846 |  |
| *CPT2* | 1 | 2 | 0.689 | >0.99 | 20 | 18 | 0.007 | >0.99 | 2.489 | 0.013 | 0.269 |  |
| *CRLF1* | 1 | 0 | 0.323 | >0.99 | 20 | 23 | 0.034 | >0.99 | 2.450 | 0.009 | 0.229 |  |
| *CYP26B1* | 0 | 0 | >0.99 | >0.99 | 34 | 47 | 0.039 | >0.99 | 1.413 | 0.036 | 0.445 |  |
| *DCXR* | 2 | 2 | 0.388 | >0.99 | 4 | 1 | 0.040 | >0.99 | 2.220 | 0.067 | 0.538 |  |
| *EGLN1* | 1 | 1 | 0.541 | >0.99 | 23 | 28 | 0.035 | >0.99 | 1.983 | 0.023 | 0.381 |  |
| *EPAS1* | 0 | 1 | >0.99 | >0.99 | 17 | 19 | 0.042 | >0.99 | 1.376 | 0.096 | 0.632 |  |
| *ERCC1* | 3 | 0 | 0.033 | >0.99 | 2 | 12 | 0.968 | >0.99 | 2.966 | 0.005 | 0.148 |  |
| *FA2H* | 0 | 0 | >0.99 | >0.99 | 13 | 10 | 0.013 | >0.99 | 1.870 | 0.023 | 0.381 |  |
| *FAS* | 0 | 0 | >0.99 | >0.99 | 4 | 0 | 0.011 | >0.99 | 1.969 | <0.001 | <0.001 |  |
| *FOXRED1* | 2 | 4 | 0.627 | >0.99 | 37 | 52 | 0.037 | >0.99 | 1.836 | 0.156 | 0.791 |  |
| *GALC* | 0 | 3 | >0.99 | >0.99 | 42 | 61 | 0.038 | >0.99 | 1.418 | 0.163 | 0.794 |  |
| *GCH1* | 0 | 1 | >0.99 | >0.99 | 6 | 3 | 0.036 | >0.99 | 1.446 | 0.081 | 0.592 |  |
| *GLIS3* | 7 | 8 | 0.177 | >0.99 | 46 | 65 | 0.023 | >0.99 | 3.147 | 0.028 | 0.393 |  |
| *GLYCTK* | 2 | 4 | 0.627 | >0.99 | 22 | 26 | 0.032 | >0.99 | 1.895 | 0.130 | 0.713 |  |
| *GNPTG* | 3 | 4 | 0.404 | >0.99 | 33 | 41 | 0.016 | >0.99 | 2.586 | 0.038 | 0.445 |  |
| *GORAB* | 0 | 0 | >0.99 | >0.99 | 20 | 16 | 0.003 | >0.99 | 2.523 | 0.003 | 0.098 |  |
| *GP1BB* | 1 | 1 | 0.541 | >0.99 | 4 | 1 | 0.040 | >0.99 | 1.932 | <0.001 | <0.001 |  |
| *GRPR* | 0 | 0 | >0.99 | >0.99 | 4 | 1 | 0.040 | >0.99 | 1.399 | <0.001 | <0.001 |  |
| *IDH2* | 0 | 0 | >0.99 | >0.99 | 15 | 12 | 0.010 | >0.99 | 2.003 | 0.012 | 0.260 |  |
| *IFITM5* | 0 | 3 | >0.99 | >0.99 | 20 | 23 | 0.034 | >0.99 | 1.467 | 0.132 | 0.717 |  |
| *IGLL1* | 0 | 2 | >0.99 | >0.99 | 37 | 45 | 0.009 | >0.99 | 2.059 | 0.066 | 0.538 |  |
| *IL21R* | 0 | 0 | >0.99 | >0.99 | 18 | 19 | 0.027 | >0.99 | 1.576 | 0.027 | 0.393 |  |
| *IL2RA* | 0 | 0 | >0.99 | >0.99 | 5 | 2 | 0.039 | >0.99 | 1.408 | <0.001 | <0.001 |  |
| *IL4R* | 0 | 2 | >0.99 | >0.99 | 15 | 10 | 0.004 | >0.99 | 2.428 | 0.015 | 0.285 |  |
| *ITGB4* | 2 | 2 | 0.388 | >0.99 | 83 | 99 | <0.001 | 0.152 | 5.094 | <0.001 | <0.001 |  |
| *JAG1* | 0 | 0 | >0.99 | >0.99 | 45 | 57 | 0.007 | >0.99 | 2.178 | 0.003 | 0.114 |  |
| *KIF5A* | 0 | 0 | >0.99 | >0.99 | 6 | 3 | 0.036 | >0.99 | 1.446 | 0.011 | 0.246 |  |
| *KRT14* | 0 | 1 | >0.99 | >0.99 | 8 | 5 | 0.028 | >0.99 | 1.553 | 0.020 | 0.350 |  |
| *LMF1* | 4 | 1 | 0.040 | >0.99 | 55 | 141 | 0.921 | >0.99 | 2.833 | 0.039 | 0.445 |  |
| *LRTOMT* | 0 | 3 | >0.99 | >0.99 | 15 | 16 | 0.044 | >0.99 | 1.361 | 0.169 | 0.794 |  |
| *MAT1A* | 0 | 0 | >0.99 | >0.99 | 20 | 22 | 0.026 | >0.99 | 1.588 | 0.016 | 0.306 |  |
| *MIB1* | 7 | 4 | 0.032 | >0.99 | 4 | 11 | 0.765 | >0.99 | 3.108 | 0.018 | 0.326 |  |
| *MTO1* | 1 | 1 | 0.541 | >0.99 | 18 | 20 | 0.036 | >0.99 | 1.981 | 0.032 | 0.423 |  |
| *MYBPC3* | 1 | 4 | 0.858 | >0.99 | 60 | 83 | 0.007 | >0.99 | 2.276 | 0.081 | 0.592 |  |
| *NDUFS8* | 0 | 0 | >0.99 | >0.99 | 9 | 6 | 0.024 | >0.99 | 1.613 | 0.057 | 0.520 |  |
| *NELFA* | 1 | 2 | 0.689 | >0.99 | 8 | 5 | 0.028 | >0.99 | 1.876 | 0.090 | 0.618 |  |
| *NODAL* | 0 | 0 | >0.99 | >0.99 | 18 | 19 | 0.027 | >0.99 | 1.576 | 0.023 | 0.381 |  |
| *NRXN1* | 0 | 1 | >0.99 | >0.99 | 47 | 71 | 0.045 | >0.99 | 1.349 | 0.039 | 0.445 |  |
| *OTOA* | 0 | 2 | >0.99 | >0.99 | 15 | 15 | 0.032 | >0.99 | 1.500 | 0.192 | 0.794 |  |
| *PAK3* | 0 | 0 | >0.99 | >0.99 | 4 | 1 | 0.040 | >0.99 | 1.399 | <0.001 | <0.001 |  |
| *PCK1* | 0 | 0 | >0.99 | >0.99 | 9 | 4 | 0.007 | >0.99 | 2.170 | 0.002 | 0.090 |  |
| *PDE4D* | 0 | 0 | >0.99 | >0.99 | 18 | 18 | 0.019 | >0.99 | 1.715 | 0.015 | 0.289 |  |
| *PDE6B* | 2 | 1 | 0.245 | >0.99 | 44 | 66 | 0.048 | >0.99 | 2.543 | 0.015 | 0.291 |  |
| *PDSS2* | 0 | 0 | >0.99 | >0.99 | 11 | 10 | 0.044 | >0.99 | 1.361 | 0.023 | 0.381 |  |
| *PLA2G6* | 2 | 2 | 0.388 | >0.99 | 15 | 11 | 0.006 | >0.99 | 3.027 | 0.002 | 0.069 |  |
| *PON1* | 3 | 0 | 0.033 | >0.99 | 2 | 14 | 0.983 | >0.99 | 2.959 | 0.003 | 0.098 |  |
| *PPIB* | 1 | 1 | 0.541 | >0.99 | 5 | 2 | 0.039 | >0.99 | 1.942 | 0.011 | 0.247 |  |
| *PRMT9* | 0 | 3 | >0.99 | >0.99 | 11 | 7 | 0.011 | >0.99 | 1.969 | 0.090 | 0.618 |  |
| *PTCH1* | 0 | 1 | >0.99 | >0.99 | 37 | 46 | 0.011 | >0.99 | 1.958 | 0.006 | 0.181 |  |
| *PTPN13* | 5 | 1 | 0.015 | >0.99 | 19 | 53 | 0.889 | >0.99 | 3.689 | 0.007 | 0.193 |  |
| *PYCR1* | 4 | 1 | 0.040 | >0.99 | 9 | 23 | 0.752 | >0.99 | 2.921 | 0.009 | 0.240 |  |
| *RAF1* | 0 | 3 | >0.99 | >0.99 | 9 | 7 | 0.040 | >0.99 | 1.397 | 0.153 | 0.783 |  |
| *RASSF1* | 10 | 6 | 0.012 | >0.99 | 0 | 5 | >0.99 | >0.99 | 3.832 | 0.008 | 0.220 |  |
| *RDX* | 1 | 0 | 0.323 | >0.99 | 12 | 11 | 0.037 | >0.99 | 2.418 | 0.001 | 0.068 |  |
| *REN* | 0 | 0 | >0.99 | >0.99 | 12 | 11 | 0.037 | >0.99 | 1.435 | 0.013 | 0.269 |  |
| *RGS9* | 0 | 2 | >0.99 | >0.99 | 12 | 5 | 0.001 | >0.99 | 2.888 | 0.005 | 0.142 |  |
| *RLIM* | 0 | 0 | >0.99 | >0.99 | 14 | 12 | 0.018 | >0.99 | 1.750 | 0.004 | 0.137 |  |
| *RRM2B* | 0 | 0 | >0.99 | >0.99 | 8 | 2 | 0.003 | >0.99 | 2.581 | <0.001 | <0.001 |  |
| *SAG* | 1 | 2 | 0.689 | >0.99 | 15 | 15 | 0.032 | >0.99 | 1.823 | 0.107 | 0.645 |  |
| *SCN3B* | 0 | 0 | >0.99 | >0.99 | 12 | 7 | 0.005 | >0.99 | 2.275 | 0.001 | 0.047 |  |
| *SCNN1G* | 0 | 0 | >0.99 | >0.99 | 31 | 41 | 0.033 | >0.99 | 1.485 | 0.024 | 0.388 |  |
| *SEMA4A* | 1 | 2 | 0.689 | >0.99 | 17 | 17 | 0.023 | >0.99 | 1.967 | 0.094 | 0.628 |  |
| *SGCA* | 0 | 0 | >0.99 | >0.99 | 36 | 51 | 0.042 | >0.99 | 1.374 | 0.043 | 0.449 |  |
| *SH2B3* | 0 | 1 | >0.99 | >0.99 | 18 | 19 | 0.027 | >0.99 | 1.576 | 0.068 | 0.538 |  |
| *SLC25A15* | 0 | 0 | >0.99 | >0.99 | 18 | 17 | 0.014 | >0.99 | 1.865 | 0.010 | 0.246 |  |
| *SLC3A1* | 2 | 3 | 0.518 | >0.99 | 33 | 44 | 0.030 | >0.99 | 2.092 | 0.073 | 0.555 |  |
| *SNRNP200* | 0 | 0 | >0.99 | >0.99 | 21 | 18 | 0.004 | >0.99 | 2.405 | 0.002 | 0.069 |  |
| *SOX18* | 0 | 0 | >0.99 | >0.99 | 16 | 16 | 0.027 | >0.99 | 1.572 | 0.025 | 0.393 |  |
| *SPECC1L* | 0 | 1 | >0.99 | >0.99 | 34 | 46 | 0.032 | >0.99 | 1.496 | 0.093 | 0.628 |  |
| *SQSTM1* | 0 | 0 | >0.99 | >0.99 | 16 | 18 | 0.050 | >0.99 | 1.305 | 0.038 | 0.445 |  |
| *SYP* | 0 | 0 | >0.99 | >0.99 | 3 | 0 | 0.033 | >0.99 | 1.476 | <0.001 | <0.001 |  |
| *TGFB2* | 0 | 0 | >0.99 | >0.99 | 6 | 2 | 0.016 | >0.99 | 1.786 | 0.010 | 0.246 |  |
| *TNFRSF13B* | 21 | 17 | 0.003 | >0.99 | 24 | 55 | 0.683 | >0.99 | 5.341 | 0.003 | 0.104 |  |
| *TXNRD2* | 1 | 0 | 0.323 | >0.99 | 13 | 5 | 0.001 | 0.802 | 4.232 | <0.001 | <0.001 |  |
| *UBL4A* | 0 | 0 | >0.99 | >0.99 | 3 | 0 | 0.033 | >0.99 | 1.476 | <0.001 | <0.001 |  |
| *UBR1* | 3 | 0 | 0.033 | >0.99 | 30 | 78 | 0.871 | >0.99 | 3.011 | 0.001 | 0.047 |  |
| *VCP* | 0 | 0 | >0.99 | >0.99 | 3 | 0 | 0.033 | >0.99 | 1.476 | <0.001 | <0.001 |  |
| *ZBTB40* | 3 | 0 | 0.033 | >0.99 | 5 | 10 | 0.561 | >0.99 | 3.202 | 0.003 | 0.111 |  |
| *ZFY* | 0 | 0 | >0.99 | >0.99 | 3 | 0 | 0.033 | >0.99 | 1.476 | <0.001 | <0.001 |  |
| *ZMYM6* | 0 | 0 | >0.99 | >0.99 | 11 | 9 | 0.029 | >0.99 | 1.539 | 0.018 | 0.321 |  |
| *ZNF469* | 0 | 1 | >0.99 | >0.99 | 182 | 321 | 0.017 | >0.99 | 1.774 | 0.055 | 0.514 |  |
| **The risk genes identified for SGA with poor prognosis based on the gene burden test in the comparison of SGA with poor prognosis (case, n=88) vs. SGA without poor prognosis (control, n=312)** | | | | | | | | | | | | |
| **Gene** | **PTV** | | | | **MIS** | | | | **Risk Score** | **Permutation P value** | **FDR^3^** |  |
|  | **The number of samples in case** | **The number of samples in control** | **P value** | **FDR^1^** | **The number of samples in case** | **The number of samples in control** | **P value** | **FDR^2^** |  |  |  |  |
| *ADIPOQ* | 0 | 0 | >0.99 | >0.99 | 8 | 4 | 0.001 | >0.99 | 3.025 | <0.001 | <0.001 |  |
| *AFF2* | 0 | 1 | >0.99 | >0.99 | 3 | 1 | 0.035 | >0.99 | 1.458 | 0.167 | 0.731 |  |
| *AR* | 0 | 0 | >0.99 | >0.99 | 9 | 9 | 0.007 | >0.99 | 2.159 | 0.002 | 0.188 |  |
| *ATXN2* | 0 | 3 | >0.99 | >0.99 | 8 | 10 | 0.025 | >0.99 | 1.597 | 0.153 | 0.731 |  |
| *BBS9* | 0 | 0 | >0.99 | >0.99 | 5 | 3 | 0.015 | >0.99 | 1.829 | 0.016 | 0.687 |  |
| *BCHE* | 2 | 0 | 0.048 | >0.99 | 0 | 9 | >0.99 | >0.99 | 2.638 | 0.049 | 0.731 |  |
| *BIN1* | 0 | 0 | >0.99 | >0.99 | 2 | 0 | 0.048 | >0.99 | 1.319 | <0.001 | <0.001 |  |
| *BLOC1S6* | 0 | 0 | >0.99 | >0.99 | 2 | 0 | 0.048 | >0.99 | 1.319 | <0.001 | <0.001 |  |
| *BRAF* | 0 | 0 | >0.99 | >0.99 | 10 | 13 | 0.015 | >0.99 | 1.833 | 0.013 | 0.687 |  |
| *CARD14* | 1 | 2 | 0.526 | >0.99 | 5 | 5 | 0.046 | >0.99 | 1.897 | 0.110 | 0.731 |  |
| *CLMP* | 0 | 0 | >0.99 | >0.99 | 3 | 1 | 0.035 | >0.99 | 1.458 | 0.037 | 0.731 |  |
| *COG1* | 2 | 0 | 0.048 | >0.99 | 3 | 9 | 0.513 | >0.99 | 2.928 | 0.014 | 0.687 |  |
| *COL11A1* | 0 | 0 | >0.99 | >0.99 | 9 | 11 | 0.016 | >0.99 | 1.795 | 0.015 | 0.687 |  |
| *COL18A1* | 2 | 0 | 0.048 | >0.99 | 6 | 29 | 0.825 | >0.99 | 2.721 | 0.040 | 0.731 |  |
| *CTSA* | 0 | 1 | >0.99 | >0.99 | 6 | 7 | 0.043 | >0.99 | 1.362 | 0.185 | 0.731 |  |
| *CYP1B1* | 0 | 0 | >0.99 | >0.99 | 10 | 16 | 0.037 | >0.99 | 1.427 | 0.014 | 0.687 |  |
| *CYP2C9* | 0 | 0 | >0.99 | >0.99 | 6 | 4 | 0.009 | >0.99 | 2.022 | 0.002 | 0.191 |  |
| *CYP7B1* | 0 | 0 | >0.99 | >0.99 | 4 | 2 | 0.023 | >0.99 | 1.639 | 0.026 | 0.731 |  |
| *DNAAF5* | 0 | 1 | >0.99 | >0.99 | 9 | 12 | 0.023 | >0.99 | 1.638 | 0.072 | 0.731 |  |
| *EFEMP1* | 0 | 0 | >0.99 | >0.99 | 2 | 0 | 0.048 | >0.99 | 1.319 | <0.001 | <0.001 |  |
| *EFEMP2* | 0 | 0 | >0.99 | >0.99 | 3 | 1 | 0.035 | >0.99 | 1.458 | 0.035 | 0.731 |  |
| *FANCE* | 0 | 0 | >0.99 | >0.99 | 4 | 2 | 0.023 | >0.99 | 1.639 | 0.023 | 0.731 |  |
| *FBN1* | 0 | 0 | >0.99 | >0.99 | 9 | 12 | 0.023 | >0.99 | 1.638 | 0.006 | 0.404 |  |
| *FGF10* | 0 | 0 | >0.99 | >0.99 | 4 | 2 | 0.023 | >0.99 | 1.639 | 0.022 | 0.731 |  |
| *FOXG1* | 0 | 0 | >0.99 | >0.99 | 2 | 0 | 0.048 | >0.99 | 1.319 | <0.001 | <0.001 |  |
| *FOXN1* | 1 | 0 | 0.22 | >0.99 | 8 | 12 | 0.049 | >0.99 | 2.624 | 0.010 | 0.580 |  |
| *FRMPD4* | 0 | 0 | >0.99 | >0.99 | 6 | 6 | 0.028 | >0.99 | 1.547 | 0.006 | 0.410 |  |
| *GDF6* | 0 | 0 | >0.99 | >0.99 | 4 | 3 | 0.045 | >0.99 | 1.350 | 0.006 | 0.404 |  |
| *GPHN* | 0 | 1 | >0.99 | >0.99 | 2 | 0 | 0.048 | >0.99 | 1.319 | 0.084 | 0.731 |  |
| *GRHL2* | 0 | 0 | >0.99 | >0.99 | 5 | 2 | 0.007 | >0.99 | 2.172 | 0.001 | 0.110 |  |
| *GSR* | 0 | 0 | >0.99 | >0.99 | 7 | 8 | 0.027 | >0.99 | 1.564 | 0.006 | 0.410 |  |
| *HSD11B2* | 0 | 0 | >0.99 | >0.99 | 2 | 0 | 0.048 | >0.99 | 1.319 | <0.001 | <0.001 |  |
| *HTT* | 3 | 0 | 0.01 | >0.99 | 8 | 30 | 0.627 | >0.99 | 4.171 | 0.005 | 0.398 |  |
| *IRX5* | 0 | 0 | >0.99 | >0.99 | 3 | 0 | 0.01 | >0.99 | 1.984 | <0.001 | <0.001 |  |
| *ITK* | 0 | 0 | >0.99 | >0.99 | 4 | 3 | 0.045 | >0.99 | 1.350 | 0.008 | 0.463 |  |
| *ITM2B* | 0 | 0 | >0.99 | >0.99 | 3 | 1 | 0.035 | >0.99 | 1.458 | 0.036 | 0.731 |  |
| *KCNE1* | 0 | 0 | >0.99 | >0.99 | 6 | 5 | 0.017 | >0.99 | 1.764 | 0.003 | 0.321 |  |
| *L2HGDH* | 0 | 0 | >0.99 | >0.99 | 6 | 7 | 0.043 | >0.99 | 1.362 | 0.012 | 0.647 |  |
| *MFN2* | 0 | 0 | >0.99 | >0.99 | 3 | 1 | 0.035 | >0.99 | 1.458 | 0.033 | 0.731 |  |
| *MKKS* | 0 | 0 | >0.99 | >0.99 | 5 | 5 | 0.046 | >0.99 | 1.340 | 0.049 | 0.731 |  |
| *MLH3* | 0 | 1 | >0.99 | >0.99 | 8 | 8 | 0.011 | >0.99 | 1.956 | 0.026 | 0.731 |  |
| *MYOT* | 0 | 0 | >0.99 | >0.99 | 2 | 0 | 0.048 | >0.99 | 1.319 | <0.001 | <0.001 |  |
| *NDUFV2* | 0 | 0 | >0.99 | >0.99 | 3 | 1 | 0.035 | >0.99 | 1.458 | 0.034 | 0.731 |  |
| *NLRP3* | 0 | 1 | >0.99 | >0.99 | 4 | 3 | 0.045 | >0.99 | 1.350 | 0.194 | 0.731 |  |
| *NOTCH2* | 0 | 0 | >0.99 | >0.99 | 7 | 5 | 0.006 | >0.99 | 2.218 | 0.001 | 0.108 |  |
| *NOTCH3* | 0 | 1 | >0.99 | >0.99 | 15 | 25 | 0.014 | >0.99 | 1.856 | 0.058 | 0.731 |  |
| *ORAI1* | 0 | 0 | >0.99 | >0.99 | 4 | 3 | 0.045 | >0.99 | 1.350 | 0.005 | 0.398 |  |
| *PAPSS2* | 4 | 3 | 0.045 | >0.99 | 5 | 2 | 0.007 | >0.99 | 4.872 | 0.002 | 0.233 |  |
| *PDSS2* | 0 | 0 | >0.99 | >0.99 | 4 | 3 | 0.045 | >0.99 | 1.350 | 0.006 | 0.410 |  |
| *PEX3* | 0 | 0 | >0.99 | >0.99 | 5 | 0 | <0.001 | >0.99 | 3.327 | 0.001 | 0.072 |  |
| *PLA2G5* | 0 | 0 | >0.99 | >0.99 | 2 | 0 | 0.048 | >0.99 | 1.319 | <0.001 | <0.001 |  |
| *PLA2G6* | 0 | 0 | >0.99 | >0.99 | 8 | 7 | 0.007 | >0.99 | 2.171 | 0.001 | 0.139 |  |
| *PON1* | 3 | 0 | 0.01 | >0.99 | 2 | 0 | 0.048 | >0.99 | 5.288 | <0.001 | <0.001 |  |
| *PRKCA* | 0 | 0 | >0.99 | >0.99 | 2 | 0 | 0.048 | >0.99 | 1.319 | <0.001 | <0.001 |  |
| *PUS1* | 0 | 0 | >0.99 | >0.99 | 3 | 0 | 0.01 | >0.99 | 1.984 | <0.001 | <0.001 |  |
| *RAD50* | 2 | 0 | 0.048 | >0.99 | 6 | 10 | 0.114 | >0.99 | 3.580 | 0.005 | 0.398 |  |
| *ROBO3* | 0 | 0 | >0.99 | >0.99 | 9 | 14 | 0.043 | >0.99 | 1.367 | 0.015 | 0.687 |  |
| *RYR1* | 0 | 1 | >0.99 | >0.99 | 19 | 38 | 0.023 | >0.99 | 1.643 | 0.097 | 0.731 |  |
| *SACS* | 0 | 0 | >0.99 | >0.99 | 14 | 26 | 0.033 | >0.99 | 1.475 | 0.015 | 0.687 |  |
| *SCN2A* | 0 | 0 | >0.99 | >0.99 | 3 | 0 | 0.01 | >0.99 | 1.984 | <0.001 | <0.001 |  |
| *SCN9A* | 2 | 0 | 0.048 | >0.99 | 3 | 16 | 0.829 | >0.99 | 2.720 | 0.039 | 0.731 |  |
| *SEC23B* | 1 | 0 | 0.22 | >0.99 | 5 | 4 | 0.028 | >0.99 | 2.874 | 0.005 | 0.398 |  |
| *SEMA4A* | 0 | 1 | >0.99 | >0.99 | 6 | 4 | 0.009 | >0.99 | 2.022 | 0.037 | 0.731 |  |
| *TAC3* | 0 | 0 | >0.99 | >0.99 | 2 | 0 | 0.048 | >0.99 | 1.319 | <0.001 | <0.001 |  |
| *TAF15* | 2 | 0 | 0.048 | >0.99 | 2 | 1 | 0.123 | >0.99 | 3.548 | 0.005 | 0.398 |  |
| *TBC1D24* | 4 | 2 | 0.023 | >0.99 | 2 | 17 | 0.947 | >0.99 | 3.301 | 0.028 | 0.731 |  |
| *TBX22* | 0 | 0 | >0.99 | >0.99 | 3 | 1 | 0.035 | >0.99 | 1.458 | 0.034 | 0.731 |  |
| *TOPORS* | 0 | 0 | >0.99 | >0.99 | 4 | 3 | 0.045 | >0.99 | 1.350 | 0.007 | 0.437 |  |
| *TTC8* | 0 | 1 | >0.99 | >0.99 | 6 | 4 | 0.009 | >0.99 | 2.022 | 0.032 | 0.731 |  |
| *TUBA8* | 0 | 0 | >0.99 | >0.99 | 7 | 7 | 0.018 | >0.99 | 1.752 | 0.004 | 0.370 |  |
| *TUFM* | 0 | 0 | >0.99 | >0.99 | 2 | 0 | 0.048 | >0.99 | 1.319 | <0.001 | <0.001 |  |
| *UNC13D* | 4 | 0 | 0.002 | >0.99 | 10 | 33 | 0.482 | >0.99 | 5.624 | 0.001 | 0.110 |  |
| *UVSSA* | 2 | 0 | 0.048 | >0.99 | 6 | 10 | 0.114 | >0.99 | 3.580 | 0.004 | 0.370 |  |
| *VKORC1* | 0 | 0 | >0.99 | >0.99 | 3 | 1 | 0.035 | >0.99 | 1.458 | 0.038 | 0.731 |  |
| *ZNF526* | 0 | 0 | >0.99 | >0.99 | 4 | 3 | 0.045 | >0.99 | 1.350 | 0.008 | 0.487 |  |

Abbreviations: PTV: protein-truncating variant; MIS: missense or non-synonymous variants; FDR: false discovery rate.

1: FDR of $P_{PTV}$

2: FDR of $P_{MIS}$

3: FDR of permutation P value for risk score

Table S11. The baseline information of the SGA-model generation dataset and the SGA-validation dataset.

|  | SGA-model generation dataset | | | | | SGA-validation dataset | | | | |
| --- | --- | --- | --- | --- | --- | --- | --- | --- | --- | --- |
|  | Overall  (n = 627) | SGA with poor prognosis (n = 89) | SGA without poor prognosis (n = 538) | Odds ratio(95% CI) | P value | Overall  (n = 115) | SGA with poor prognosis (n = 11) | SGA without poor prognosis  (n = 104) | Odds ratio(95% CI) | P value |
| Gestational age,mean (SD), week | 35.83 (3.36) | 35.45 (3.80) | 35.89 (3.28) | 0.96(0.9-1.03) | 0.45 | 36.46 (3.01) | 34.91 (3.51) | 36.62 (2.93) | 0.85(0.7-1.03) | 0.11 |
| Birth weight, mean (SD), g | 1,954.02 (634.15) | 1,898.20 (707.33) | 1,963.25 (621.45) | 1(1-1) | 0.47 | 2,076.13 (627.54) | 1,674.09 (782.51) | 2,118.65 (597.77) | 1(1-1) | 0.067 |
| Male (%) | 382 (60.9) | 61 (68.5) | 321 (59.7) | 0.68(0.42-1.09) | 0.11 | 68 (59.1) | 7 (63.6) | 61 (58.7) | 0.81(0.2-2.86) | >0.99 |
| Genetic factor: rare variant burden score, mean (SD) | 0.71 (1.20) | 2.27 (1.78) | 0.46 (0.83) | 2.78(2.29-3.43) | <0.001 | 1.13 (1.10) | 2.09 (1.14) | 1.03 (1.06) | 2.15(1.28-3.79) | 0.004 |
| Clinical factors: clinical abnormalities between SGA with and without poor prognosis | | | | | | | | | | |
| Neurologic (%) | 184 (29.3) | 46 (51.7) | 138 (25.7) | 3.1(1.96-4.92) | <0.001 | 21 (18.3) | 3 (27.3) | 18 (17.3) | 1.79(0.37-6.9) | 0.42 |
| Metabolic/biochemical (%) | 234 (37.3) | 48 (53.9) | 186 (34.6) | 2.22(1.41-3.5) | <0.001 | 27 (23.5) | 4 (36.4) | 23 (22.1) | 2.01(0.49-7.28) | 0.28 |
| Skeletal (%) | 27 (4.3) | 9 (10.1) | 18 (3.3) | 3.25(1.35-7.32) | 0.008 | 6 (5.2) | 0 (0) | 6 (5.8) | NA | >0.99 |
| Respiratory (%) | 191 (30.5) | 44 (49.4) | 147 (27.3) | 2.6(1.65-4.11) | <0.001 | 17 (14.8) | 3 (27.3) | 14 (13.5) | 2.41(0.49-9.53) | 0.21 |
| Allergy/immunologic/infectious (%) | 302 (48.2) | 56 (62.9) | 246 (45.7) | 2.01(1.28-3.23) | 0.003 | 41 (35.7) | 7 (63.6) | 34 (32.7) | 3.6(1.02-14.55) | 0.052 |
| Craniofacial (%) | 33 (5.3) | 11 (12.4) | 22 (4.1) | 3.31(1.49-6.96) | 0.003 | 2 (1.7) | 0 (0) | 2 (1.9) | NA | >0.99 |

Abbreviations: NA: Not Available
