# Supplementary material for: Comprehensive assessment of the genetic characteristics of small for gestational age newborns in NICU: from diagnosis of genetic disorders to prediction of prognosis
Source: Genome Med. 2023 Dec 13;15:112. doi: 10.1186/s13073-023-01268-2 (PMC10717355; doi:10.1186/s13073-023-01268-2)
Supplement: Supplementary file 2 — Additional file 2: Figure S1. Flowchart of SGA prognosis prediction model. [file 13073_2023_1268_MOESM2_ESM.pdf]

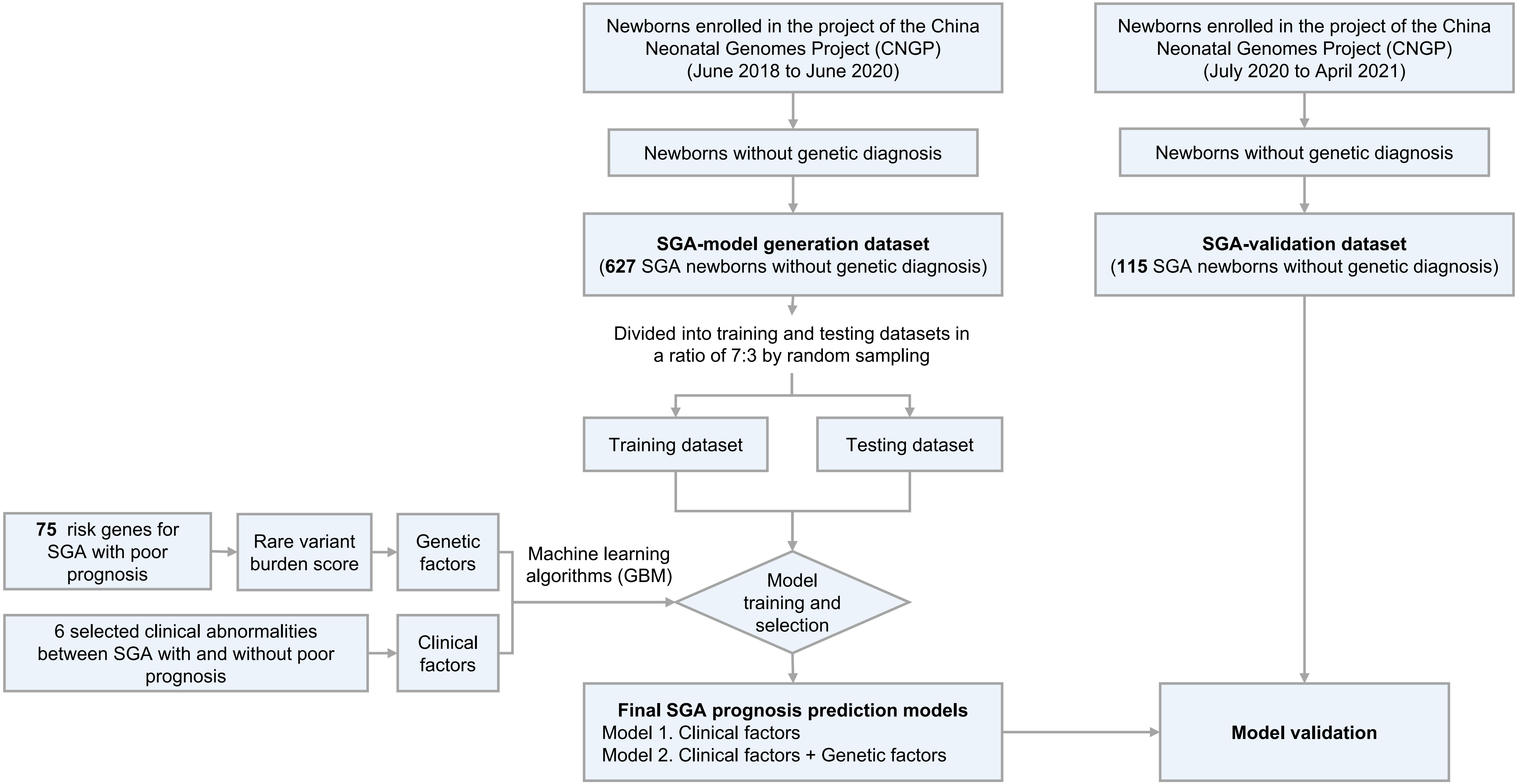
Newborns enrolled in the project of the China Neonatal Genomes Project (CNGP) (June 2018 to June 2020)
Newborns enrolled in the project of the China Neonatal Genomes Project (CNGP) (July 2020 to April 2021)
Newborns without genetic diagnosis
Newborns without genetic diagnosis
SGA-model generation dataset (627 SGA newborns without genetic diagnosis)
SGA-validation dataset (115 SGA newborns without genetic diagnosis)
Divided into training and testing datasets in a ratio of 7:3 by random sampling
Training dataset
Testing dataset
75 risk genes for SGA with poor prognosis
Rare variant burden score
Genetic factors
6 selected clinical abnormalities between SGA with and without poor prognosis
Clinical factors
Machine learning algorithms (GBM)
Model training and selection
Final SGA prognosis prediction models
Model 1. Clinical factors
Model 2. Clinical factors + Genetic factors
Model validation
